# Longitudinal Trajectories of Autonomic Maturation in Preterm Infants Using Serial 24-Hour Heart Rate Variability Monitoring

**DOI:** 10.3390/bios16100586

**Published:** 2026-10-19

**Authors:** Masashiro Sugino, Toru Kuboi, Yoko Tadatomo, Nana Kawaguchi, Takaaki Sadamura, Akiko Nakano, Kosuke Koyano, Shinji Nakamura, Ken Takahashi, Takashi Kusaka

**Affiliations:** 1 Department of Neonatology, National Hospital Organization Shikoku Medical Center for Children and Adults, Zentsuji 765-8507, Japan; kuboi517@gmail.com (T.K.); yoko_tadatomo3219@yahoo.co.jp (Y.T.); nana_maruo@yahoo.co.jp (N.K.); volttt2000@gmail.com (T.S.); nakano.akiko.ht@mail.hosp.go.jp (A.N.); 2 Department of Pediatrics, Faculty of Medicine, Kagawa University, Takamatsu 760-0016, Japan; koyano.kosuke@kagawa-u.ac.jp (K.K.); kusaka.takashi@kagawa-u.ac.jp (T.K.); 3 Division of Neonatology, Perinatal Medical Center, Jichi Medical University, Shimotsuke 329-0498, Japan; shinji980@hotmail.com; 4 Department of Pediatrics, Juntendo University Urayasu Hospital, Chiba 279-0021, Japan; kentaka@juntendo.ac.jp

**Keywords:** heart rate variability, preterm infant, autonomic nervous system, postmenstrual age, longitudinal study, neonatal intensive care unit

## Abstract

Heart rate variability (HRV) is a non-invasive measure reflecting cardiac autonomic regulation and has been used to investigate developmental changes in preterm infants. Short-term HRV recordings have demonstrated developmental changes with advancing postmenstrual age (PMA), but are highly susceptible to transient physiological conditions, limiting reproducibility. We prospectively performed serial 24 h electrocardiographic recordings in 50 preterm infants born at 24–35 weeks’ gestation from postnatal life until before 37 weeks’ PMA to characterize longitudinal HRV trajectories. A total of 879 recordings were analyzed using a 24 h single-window approach, and developmental trajectories were evaluated using linear mixed-effects models. All HRV indices increased progressively with advancing PMA. Infants born at ≥32 weeks’ gestation exhibited significantly higher HRV values after approximately 33 weeks’ PMA than those born at <32 weeks, indicating distinct maturational trajectories according to gestational age. PMA remained independently associated with multiple HRV indices after adjustment for major clinical factors, whereas body weight z-score, respiratory support, oxygen supplementation, and caffeine exposure showed little or no independent association after adjustment. These findings characterize longitudinal 24 h HRV trajectories in preterm infants and suggest that serial 24 h HRV monitoring may provide a framework for assessing developmental changes in cardiac autonomic regulation during neonatal intensive care.

## 1. Introduction

The autonomic nervous system (ANS) undergoes rapid maturation during the late fetal and early postnatal periods and plays a fundamental role in cardiovascular regulation, respiratory stability, and neurodevelopment [1,2]. Heart rate variability (HRV), which quantifies beat-to-beat fluctuations in cardiac rhythm, is a non-invasive measure reflecting cardiac autonomic regulation and has been widely used to investigate developmental changes in autonomic function in preterm infants [3,4]. Previous studies using HRV have consistently demonstrated progressive increases in both time- and frequency-domain indices with advancing postmenstrual age (PMA), changes that are considered to be associated with the development of sympathetic and parasympathetic regulation [5,6,7]. Altered HRV patterns have been associated with adverse neurodevelopmental outcomes, suggesting that HRV may have potential as a biomarker of atypical autonomic development [8,9].

Despite its clinical importance, the assessment of HRV in preterm infants remains methodologically challenging. Most previous studies have relied on short-term recordings lasting only 5–30 min [5,6,7,8,9,10]. Because HRV is highly influenced by transient physiological conditions—including feeding, sleep state, body position, handling, and routine clinical procedures—short-term recordings require strict standardization of measurement conditions and are susceptible to substantial variability [11,12,13]. Recent reviews have further highlighted considerable heterogeneity in recording protocols and analytical methods across studies, limiting reproducibility and preventing the establishment of standardized developmental reference [14].

Continuous 24 h HRV monitoring offers a practical alternative by averaging physiological fluctuations over an entire day and enables assessment during routine neonatal intensive care without strict environmental control. A previous study in preterm infants born at 28–32 weeks’ gestation demonstrated that this approach is feasible and provides highly reproducible 24 h HRV measurements during routine neonatal intensive care unit (NICU) care [15]. However, they primarily focused on reproducibility between sequential 24 h recordings and short-term developmental changes between 32 and 35 weeks’ PMA. Previous studies have demonstrated maturational increases in HRV and differences according to gestational age using both short-term and longer recordings [6,7,15]. However, longitudinal characterization using repeated 24 h recordings across a broad range of PMA during routine NICU hospitalization remains limited. In particular, the relative contributions of developmental age, somatic growth, postnatal age, and common clinical interventions to these longitudinal trajectories have not been fully characterized. This knowledge gap limits the characterization of longitudinal HRV development during routine NICU care and the identification of infants with atypical HRV trajectories.

We hypothesized that continuous 24 h HRV monitoring would characterize developmental HRV trajectories that have not been fully captured using conventional short-term recordings because of their susceptibility to transient physiological influences. We further hypothesized that these trajectories would differ according to gestational age at birth.

To test this hypothesis, we aimed to characterize longitudinal 24 h HRV trajectories from early postnatal life until approximately 36 weeks’ PMA. We further compared these trajectories according to gestational age and evaluated the independent contributions of PMA, body weight z-score, day of life, and major clinical factors to HRV maturation using linear mixed-effects models. The present study was intended to characterize longitudinal 24 h HRV trajectories rather than to establish validated clinical reference standards. Such trajectories may provide a framework for future studies aimed at establishing normative reference ranges and determining whether deviations from expected HRV trajectories are associated with adverse clinical or neurodevelopmental outcomes.

## 2. Materials and Methods

### 2.1. Participants

This prospective longitudinal observational study enrolled consecutive preterm infants born at 24–35 weeks’ gestation who were admitted to the neonatal intensive care unit (NICU) at the National Hospital Organization Shikoku Medical Center for Children and Adults, Kagawa, Japan, between November 2024 and May 2026. Infants with major congenital anomalies, hemodynamically significant congenital heart disease, grade III–IV intraventricular hemorrhage, or conditions preventing reliable electrocardiographic (ECG) recording were excluded.

The study was approved by the Institutional Review Board of the National Hospital Organization Shikoku Medical Center for Children and Adults (approval no. R05-18). Written informed consent was obtained from the parents or legal guardians of all participants before enrollment.

### 2.2. Data Acquisition and HRV Analysis

The ECG waveforms were continuously recorded using a portable three-lead ECG recorder (FAROS 180^®^, Bittium Corporation, Oulu, Finland) at a sampling frequency of 1000 Hz. Recordings were performed during routine neonatal intensive care without altering standard clinical management. Continuous 24 h ECG recordings were obtained serially from early postnatal life until approximately 36 weeks of PMA. For most infants, recordings were repeated at approximately 5-day intervals, corresponding to the battery replacement cycle of the recording device.

Raw RR interval (RRI) data were exported from the FAROS system and analyzed using Kubios HRV Scientific software (version 4.2.0; Kubios Oy, Kuopio, Finland) [16]. Automatic artifact detection and correction were applied uniformly to all recordings using the built-in Kubios correction algorithm with standardized settings. The proportion of beats subjected to artifact correction was recorded for each 24 h recording as an index of signal quality. Each recording day was treated as a single 24 h analysis window (24 h single-window approach) [14]. Recordings were included in the analysis when at least 23.5 h of analyzable data were available.

Time-domain HRV indices included the standard deviation of the normal-to-normal interval index (SDNNI), the standard deviation of normal-to-normal intervals (SDNN), and the root mean square of successive differences (RMSSD). SDNNI reflects intermediate-term variability across the recording period, SDNN reflects overall heart rate variability, and RMSSD primarily reflects parasympathetic modulation [4].

Frequency-domain analysis was performed using autoregressive spectral estimation (Burg method, model order 16). Power spectral density was calculated from resampled RRI data (4 Hz) and integrated within predefined neonatal frequency bands: very-low-frequency (VLF, 0.0033–0.04 Hz), low-frequency (LF, 0.04–0.15 Hz), and high-frequency (HF, 0.40–2.00 Hz) [4]. The upper HF boundary of 2.00 Hz was selected to account for the higher respiratory rates observed in preterm infants [15]. VLF reflects long-term regulatory mechanisms, including thermoregulatory and hormonal influences; LF reflects mixed sympathetic and parasympathetic modulation; and HF predominantly reflects respiratory-related vagal activity. Ultra-low-frequency (ULF; <0.0033 Hz) power was not included in the prespecified analysis. Although the 24 h recording duration permits assessment of ULF, its physiological determinants and frequency boundaries remain insufficiently established in neonates, with substantial methodological heterogeneity across previous neonatal HRV studies; therefore, the present analysis focused on the more commonly reported neonatal VLF, LF, and HF bands [4,14].

For each recording, a single set of 24 h HRV indices (SDNNI, SDNN, RMSSD, VLF, LF, and HF) was obtained. At each HRV assessment, body weight z-scores were calculated according to the infant’s PMA using Japanese neonatal growth reference standards [17]. Longitudinal developmental trajectories were subsequently evaluated according to PMA and gestational age.

Clinical variables were recorded for each 24 h HRV recording and analyzed as time-varying covariates. Invasive respiratory support was defined as endotracheal mechanical ventilation, whereas non-invasive respiratory support included continuous positive airway pressure (CPAP) and high-flow nasal cannula (HFNC). Oxygen supplementation was defined as the administration of supplemental oxygen irrespective of the mode of respiratory support. Catecholamine use was defined as treatment with dopamine and/or dobutamine on the day of HRV recording. Caffeine exposure was defined as any previous or ongoing caffeine therapy at the time of HRV assessment; infants who had never received caffeine during the study period served as the reference group.

### 2.3. Statistical Analysis

Statistical analyses were performed using MATLAB R2024b (MathWorks, Natick, MA, USA) and R version 4.3.2 (R Foundation for Statistical Computing, Vienna, Austria). Continuous variables are presented as median (interquartile range [IQR]) or regression coefficients with 95% confidence intervals (CI), as appropriate. Baseline characteristics were compared across the three gestational-age groups using the Kruskal–Wallis test for continuous variables and the Fisher–Freeman–Halton exact test for categorical variables. These *p*-values represent overall between-group comparisons among the three groups. All statistical tests were two-sided, and *p*-values <0.05 were considered statistically significant.

Longitudinal changes in HRV were evaluated using linear mixed-effects models with infant-specific random intercepts to account for repeated measurements within individuals. Separate models were constructed for each HRV index (SDNNI, SDNN, RMSSD, VLF, LF, and HF).

To investigate the independent associations of biological maturation, relative growth status, and postnatal age with HRV, additional mixed-effects models included PMA, PMA-specific body weight z-score, and day of life (DOL) as fixed effects. Regression coefficients (β) with 95% CI were estimated to quantify the independent association of each predictor with the corresponding HRV index.

To evaluate potential clinical confounders, multivariable mixed-effects models were constructed, including PMA, invasive respiratory support, non-invasive respiratory support, oxygen supplementation, catecholamine use, and caffeine exposure as fixed effects, with infant identification number included as a random intercept. Regression coefficients (β), 95% CIs, and corresponding *p*-values were reported.

For descriptive visualization, weekly mean HRV values with 95% CIs were plotted according to PMA. Infants were stratified into three gestational-age groups (<28 weeks, 28–31 weeks, and ≥32 weeks), and individual longitudinal trajectories were visualized using spaghetti plots.

Because PMA coverage differed among gestational-age groups, formal between-group comparisons were restricted to the overlapping period from 33 to 36 weeks’ PMA. These models included PMA and gestational-age group (<32 vs. ≥32 weeks) as fixed effects and infant ID as a random intercept.

## 3. Results

### 3.1. Participant Characteristics

Fifty preterm infants met the inclusion criteria for the analysis. Their baseline characteristics are summarized in Table 1. The median gestational age at birth was 32.3 weeks (IQR, 30.3–34.0), and the median birth weight was 1486 g (IQR, 1114–1908). Infants were classified into three gestational-age groups: <28 weeks (n = 5), 28–31 weeks (n = 15), and ≥32 weeks (n = 30).

A total of 879 serial 24 h HRV recordings obtained between 26 and 36 weeks’ PMA were included in the longitudinal analyses, including 171, 401, and 307 recordings from the <28, 28–31, and ≥32-week groups, respectively. The infant-level recording coverage and the distribution of analyzed recordings across PMA weeks are summarized in Supplementary Tables S1 and S3 (the minimal dataset). Regarding signal quality, the median proportion of artifact-corrected beats across the 879 recordings was 1.60% (IQR, 1.33–2.00%; range, 0.43–4.97%). Of these, 654 recordings (74.4%) had <2% corrected beats.

### 3.2. Longitudinal Trajectories of HRV Indices

**Figure 1:**
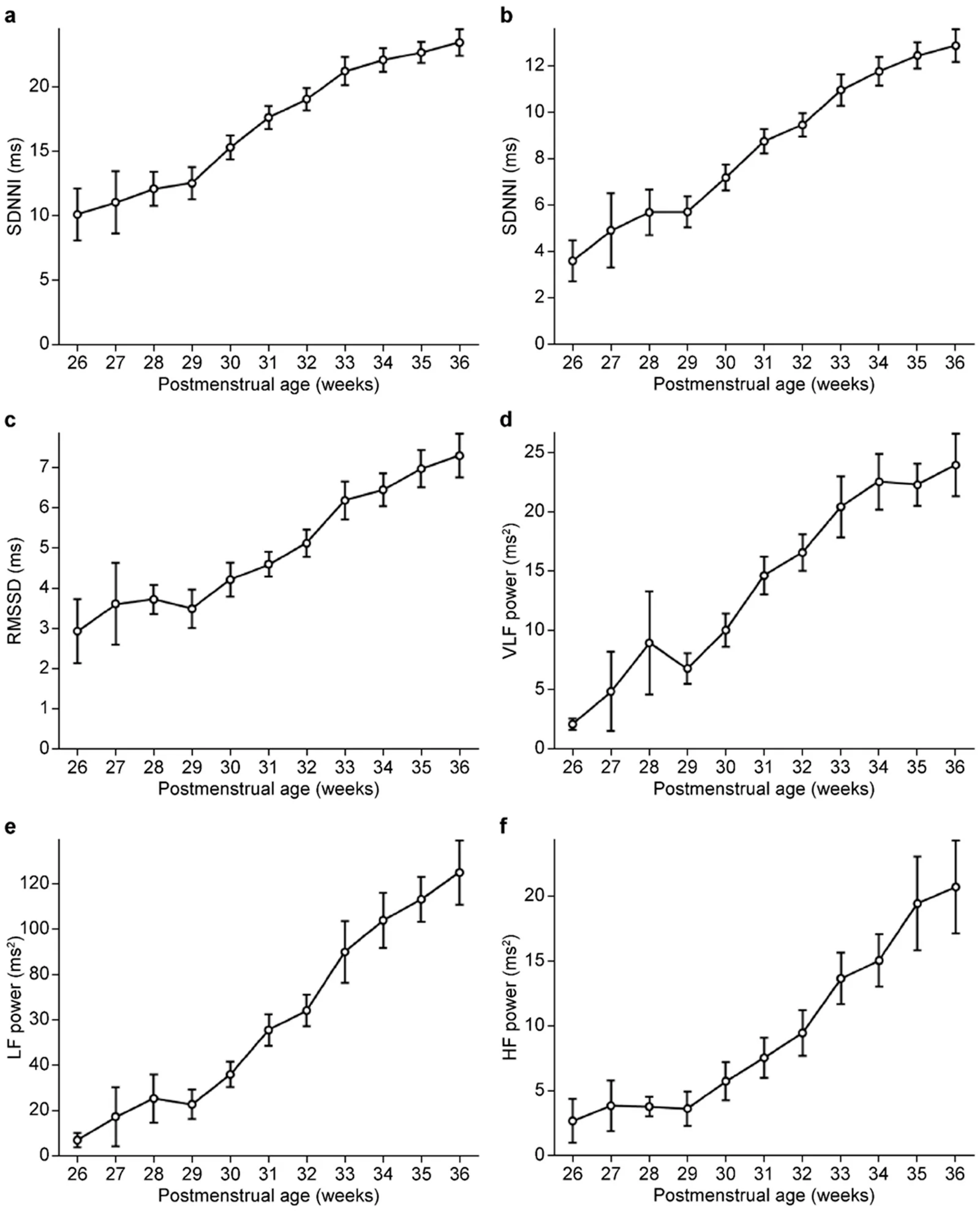
Longitudinal trajectories of 24 h heart rate variability in preterm infants. Mean values of six HRV indices obtained from serial 24 h electrocardiographic recordings in all preterm infants. Symbols represent the weekly mean values according to PMA, and error bars indicate the 95% confidence intervals. The solid lines connect the weekly mean values for visual guidance. Panels show (**a**) SDNNI, (**b**) SDNN, (**c**) RMSSD, (**d**) VLF power, (**e**) LF power, and (**f**) HF power. All HRV indices increased progressively with advancing PMA.

**Figure 2:**
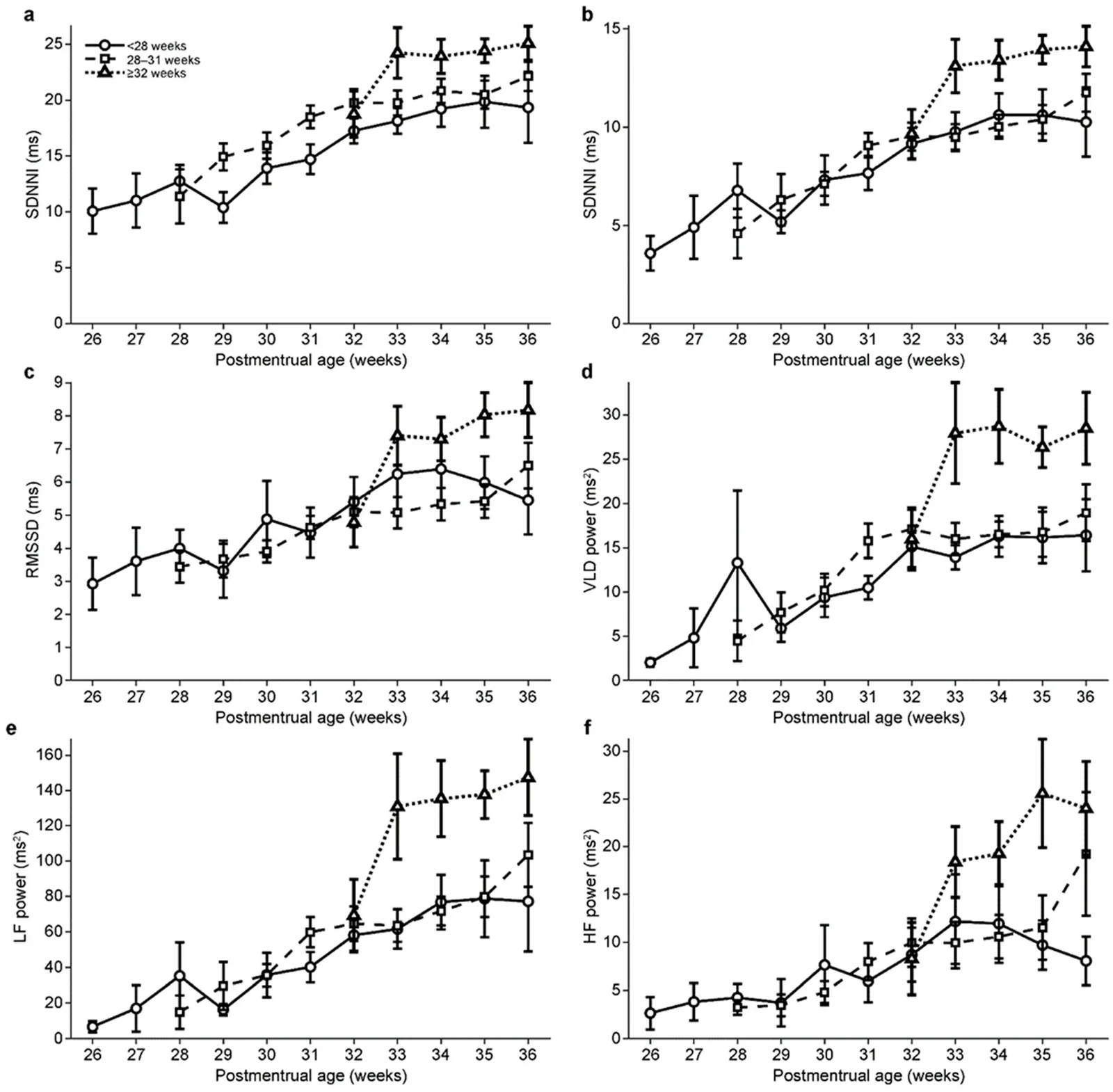
Longitudinal trajectories of 24 h heart rate variability according to gestational age. Mean trajectories of six HRV indices stratified by gestational age (<28 weeks, 28–31 weeks, and ≥32 weeks). Symbols represent weekly mean values according to PMA, and error bars indicate the 95% confidence intervals. Panels show (**a**) SDNNI, (**b**) SDNN, (**c**) RMSSD, (**d**) VLF power, (**e**) LF power, and (**f**) HF power. Infants born at ≥32 weeks showed consistently higher HRV values after approximately 33 weeks’ PMA than infants born at earlier gestational ages.

The longitudinal trajectories of HRV indices are shown in Figure 1 and Figure 2. Figure 1 presents the overall trajectories, whereas Figure 2 shows the trajectories stratified by gestational age. In the overall cohort, all time-domain (SDNNI, SDNN, and RMSSD) and frequency-domain (VLF, LF, and HF) HRV indices increased progressively with advancing PMA, consistent with progressive developmental changes in cardiac autonomic regulation (Figure 1).

When stratified according to gestational age, distinct developmental trajectories were observed (Figure 2). Infants born at <28 weeks and 28–31 weeks showed gradual increases in all HRV indices throughout the observation period. In contrast, infants born at ≥32 weeks exhibited a steeper increase beginning at approximately 33 weeks’ PMA and maintained higher HRV values thereafter than the other two groups. These differences were consistently observed across both time-domain and frequency-domain HRV indices. Because the available PMA range differed according to gestational age at birth, these group-specific trajectories are presented primarily for descriptive purposes. Formal between-group comparisons were therefore restricted to the overlapping period from 33 to 36 weeks’ PMA.

To further evaluate this apparent divergence, an additional linear mixed-effects analysis restricted to measurements obtained between 33 and 36 weeks’ PMA was performed (Supplementary Table S2). After adjustment for PMA, infants born at ≥32 weeks exhibited significantly higher SDNNI, SDNN, RMSSD, VLF, and LF values than those born at <32 weeks, whereas HF showed a similar trend but did not reach a statistical significance (*p* = 0.057).

Individual longitudinal trajectories are presented in Supplementary Figures S1 and S2. Although inter-individual variability was observed, the overall increase in HRV with advancing PMA was consistently present across most infants, indicating that the population-level trajectories were not driven by a small number of participants.

### 3.3. Associations of PMA, Body Weight Z-Score, and Day of Life with HRV Indices

The independent associations of PMA, body weight z-score, and day of life (DOL) with HRV indices were evaluated using linear mixed-effects models (Table 2).

PMA was independently associated with all HRV indices, including SDNNI (β = 1.74, 95% CI 1.17–2.32, *p* < 0.001), SDNN (β = 1.21, 95% CI 0.83–1.59, *p* < 0.001), RMSSD (β = 0.68, 95% CI 0.40–0.96, *p* < 0.001), VLF power (β = 3.10, 95% CI 1.69–4.50, *p* < 0.001), LF power (β = 22.60, 95% CI 14.85–30.35, *p* < 0.001), and HF power (β = 3.89, 95% CI 1.81–5.97, *p* < 0.001).

Body weight z-score was not independently associated with any HRV parameter after adjustment for PMA and DOL. In contrast, DOL showed significant negative associations with SDNNI, SDNN, RMSSD, VLF power, and LF power, whereas no significant association was observed for HF power.

### 3.4. Associations Between Clinical Factors and HRV Indices

To evaluate the associations of clinical factors with HRV indices, multivariable linear mixed-effects models including PMA, invasive respiratory support, non-invasive respiratory support, oxygen supplementation, catecholamine use, and caffeine exposure were performed (Table 3).

After adjustment for these clinical factors, PMA remained independently associated with SDNNI (β = 0.55, 95% CI 0.37–0.73, *p* < 0.001), SDNN (β = 0.38, 95% CI 0.27–0.50, *p* < 0.001), RMSSD (β = 0.17, 95% CI 0.09–0.25, *p* < 0.001), LF power (β = 4.82, 95% CI 2.65–6.98, *p* < 0.001), and HF power (β = 0.76, 95% CI 0.26–1.26, *p* = 0.003), whereas the association with VLF power did not reach a statistical significance (β = 0.40, 95% CI −0.01–0.82, *p* = 0.058).

Invasive respiratory support was independently associated with lower SDNN (β = −1.23, 95% CI −2.14–−0.33, *p* = 0.008) and RMSSD (β = −0.93, 95% CI −1.60–−0.26, *p* = 0.006), whereas non-invasive respiratory support and oxygen supplementation were not independently associated with any HRV index.

Catecholamine use was consistently associated with lower HRV, showing significant negative associations with SDNNI (β = −1.26, 95% CI −2.18–−0.34, *p* = 0.007), SDNN (β = −1.13, 95% CI −1.70–−0.55, *p* < 0.001), RMSSD (β = −0.59, 95% CI −1.01–−0.16, *p* = 0.006), VLF power (β = −2.55, 95% CI −4.69–−0.40, *p* = 0.020), and LF power (β = −15.43, 95% CI −26.47–−4.38, *p* = 0.006), whereas no significant association was observed with HF power.

Caffeine exposure was independently associated with higher SDNNI (β = 1.59, 95% CI 0.30–2.87, *p* = 0.015); however, showed no significant associations with the remaining HRV indices.

Overall, PMA remained independently associated with most HRV indices after adjustment for the included clinical factors, whereas catecholamine use was consistently associated with lower HRV across multiple time- and frequency-domain indices. In contrast, respiratory interventions and caffeine exposure showed limited independent associations with the observed developmental trajectories.

## 4. Discussion

In this longitudinal study, we characterized longitudinal 24 h HRV trajectories throughout NICU hospitalization. Consistent with previous studies, HRV increased progressively with advancing PMA, and infants born at earlier gestational ages exhibited lower HRV at comparable PMAs. These findings therefore confirm and extend previously reported maturational patterns rather than represent entirely novel observations. The principal additional contribution of the present study is the demonstration, using dense serial 24 h recordings and longitudinal mixed-effects modeling, that PMA remained independently associated with HRV after accounting for somatic growth, postnatal age, and major clinical factors.

The progressive increase in HRV across PMA is consistent with developmental changes in cardiac autonomic regulation during the preterm period [7,18]. However, HRV is not a direct measure of ANS maturation and may also be influenced by illness severity, respiratory status, medications, sleep state, feeding, and other physiological conditions. Although previous studies using conventional short-term HRV recordings have suggested similar developmental trends, those measurements are inherently susceptible to transient physiological influences, including feeding, sleep state, handling, and routine clinical procedures, limiting their reproducibility for longitudinal assessment [11,12,13,14]. In contrast, our study employed repeated 24 h HRV recordings from early postnatal life until approximately 36 weeks’ PMA, enabling physiological fluctuations to be averaged over an entire day under routine neonatal intensive care conditions [15]. This approach enabled robust characterization of longitudinal 24 h HRV trajectories while reducing the influence of transient physiological fluctuations that can affect conventional short-term recordings.

The second principal finding was that longitudinal HRV trajectories differed according to gestational age. Although HRV increased with PMA in all groups, infants born at ≥32 weeks exhibited significantly higher HRV values after approximately 33 weeks’ PMA than infants born before 32 weeks. These findings suggest that longitudinal HRV development may be associated not only with current PMA but also with gestational age at birth [19,20]. Earlier exposure to the extrauterine environment may alter the normal developmental trajectory of autonomic regulation through prolonged physiological stress, repeated clinical procedures, respiratory instability, or inflammatory exposure [18]. Although the underlying mechanisms remain uncertain, our findings indicate that gestational age remained associated with HRV even after adjustment for PMA.

The third principal finding was that PMA remained independently associated with HRV after adjustment for body weight z-score, DOL, and major clinical variables. Body weight z-score was not independently associated with the HRV indices after accounting for PMA and DOL. Interestingly, DOL showed negative associations with several HRV indices after adjustment for PMA. These findings indicate that developmental age is closely associated with longitudinal HRV changes; however, the relative importance of PMA, DOL, and other predictors cannot be inferred by directly comparing unstandardized regression coefficients because these variables were measured on different scales.

Further analysis of the adjusted models showed that respiratory support was not uniformly associated with HRV across all indices. Invasive respiratory support was independently associated with lower SDNN and RMSSD, whereas non-invasive respiratory support and oxygen supplementation showed no consistent associations. Mechanical ventilation may influence HRV through alterations in intrathoracic pressure and cardiopulmonary interactions [21]; however, the need for invasive respiratory support is also closely related to underlying illness severity [22]. Therefore, the observed associations may reflect the effects of mechanical ventilation, greater clinical severity, or both. PMA nevertheless remained independently associated with most HRV indices after adjustment for these clinical factors. Catecholamine use was associated with lower HRV across multiple indices, likely reflecting greater cardiovascular instability or illness severity in infants requiring vasoactive support, although a direct pharmacological effect of catecholamines on autonomic regulation cannot be excluded [23]. Importantly, these associations are subject to confounding by indication. Infants receiving invasive respiratory support or catecholamines were likely to have greater underlying illness severity and cardiorespiratory instability. Therefore, the observed reductions in HRV cannot be attributed causally to these treatments themselves.

Caffeine exposure showed only limited associations with HRV after multivariable adjustment. Given that caffeine directly influences autonomic regulation and cardiorespiratory control [24], its effects on HRV may depend on the actual systemic exposure rather than on treatment history alone. Our previous study demonstrated substantial interindividual variability in serum caffeine concentrations among preterm infants, with some infants exhibiting concentrations exceeding the recommended therapeutic range despite receiving standard weight-adjusted doses [25]. Therefore, the absence of consistent associations in this study may reflect the inability of a binary exposure variable to capture differences in systemic caffeine exposure. Future studies incorporating serum caffeine concentrations together with serial 24 h HRV monitoring may clarify whether caffeine exerts concentration-dependent effects on HRV.

Collectively, these findings indicate that longitudinal changes in HRV were independently associated with PMA, while invasive respiratory support, catecholamine use, and other indicators of clinical severity may also influence specific HRV indices.

The major strength of this study was the use of serial 24 h HRV monitoring throughout hospitalization. Unlike conventional short-term recordings, continuous monitoring minimizes the influence of temporary physiological fluctuations and allows repeated evaluation without strict control of environmental conditions [15]. Furthermore, longitudinal mixed-effects modeling enabled evaluation of repeated measurements while accounting for within-subject correlations and major clinical confounders, providing a more robust assessment of longitudinal HRV changes than cross-sectional analyses.

This study had some limitations. First, this was a single-center study with a relatively limited sample size, particularly in the extremely preterm subgroup. Although repeated measurements were accounted for using mixed-effects models, the large number of recordings cannot compensate for the small number of independent infants in this subgroup. Accordingly, the gestational-age-specific trajectories, particularly those of the <28-week group, should be interpreted cautiously and require confirmation in larger cohorts. Second, although several important clinical variables were adjusted for, residual confounding by unmeasured factors, including infection severity, nutritional status, medication timing, and sleep state, cannot be excluded. PMA coverage differed among gestational-age groups because measurements could only be obtained after birth. Therefore, the gestational-age-stratified trajectories across the full PMA range should be interpreted descriptively, and formal between-group comparisons were restricted to the overlapping period of 33–36 weeks’ PMA. Third, because this study focused on developmental trajectories during hospitalization, the relationship between neonatal HRV maturation and long-term neurodevelopmental outcomes remains unknown. Future multicenter studies with larger cohorts and longitudinal neurodevelopmental follow-up will be required to establish clinically useful reference standards.

## 5. Conclusions

This study characterized longitudinal trajectories of 24 h HRV in preterm infants from early postnatal life to approximately 36 weeks PMA. Consistent with previous studies, HRV increased with advancing PMA and differed according to gestational age at birth. PMA remained independently associated with most HRV indices after adjustment for major clinical factors. These densely sampled 24 h data provide a framework for future validation of developmental HRV reference trajectories. Serial 24 h HRV monitoring may have potential utility for assessing developmental changes in cardiac autonomic regulation; however, larger multicenter studies are required to establish validated reference ranges and to determine whether deviations from expected HRV trajectories are associated with adverse neurodevelopmental outcomes.

## Figures and Tables

**Table 1:** Background characteristics of study infants by gestational age group.

Characteristic	All (n = 50)	<28 Weeks (n = 5)	28–31 Weeks (n = 15)	≥32 Weeks (n = 30)	*p*-Value
Gestational age, weeks	32.3 (30.3–34.0)	26.3 (25.9–26.9)	30.3 (29.5–30.4)	33.8 (32.9–34.7)	—
Birth weight, g	1486 (1114–1908)	822 (655–929)	1132 (1009–1473)	1876 (1422–2056)	<0.001
Birth weight z-score	−0.7 (−1.9–0.1)	−0.5 (−1.1–0.3)	−0.9 (−1.9–0.3)	−0.6 (−1.9–−0.1)	0.744
Male sex	24/50 (48%)	2/5 (40%)	6/15 (40%)	16/30 (53%)	0.637
Apgar score at 1 min	6.0 (4.0–8.0)	3.0 (2.0–3.5)	4.0 (2.2–5.0)	7.5 (6.0–8.0)	<0.001
Apgar score at 5 min	8.0 (7.0–9.0)	7.0 (5.8–7.2)	7.0 (5.2–8.0)	9.0 (8.0–9.0)	<0.001
Chorioamnionitis	9/40 (23%)	2/5 (40%)	6/14 (43%)	1/21 (5%)	0.015
Antenatal corticosteroids ≥ 1 dose	16/50 (32%)	3/5 (60%)	7/15 (47%)	6/30 (20%)	0.069
Treated patent ductus arteriosus (cyclooxygenase inhibitors)	3/50 (6%)	3/5 (60%)	0/15 (0%)	0/30 (0%)	<0.001
Inotrope use in the acute phase	46/50 (92%)	5/5 (100%)	15/15 (100%)	26/30 (87%)	0.396
Surfactant treatment	33/50 (66%)	5/5 (100%)	15/15 (100%)	13/30 (43%)	<0.001
Caffeine therapy	36/50 (72%)	5/5 (100%)	15/15 (100%)	16/30 (53%)	<0.001
Early-onset sepsis	1/50 (2%)	1/5 (20%)	0/15 (0%)	0/30 (0%)	0.100
Late-onset sepsis	1/50 (2%)	0/5 (0%)	1/15 (7%)	0/30 (0%)	0.400
Intraventricular hemorrhage ≥ grade 1	3/50 (6%)	1/5 (20%)	2/15 (13%)	0/30 (0%)	0.073
Cystic periventricular leukomalacia	0/50 (0%)	0/5 (0%)	0/15 (0%)	0/30 (0%)	1.000
Necrotizing enterocolitis ≥ stage 2	0/50 (0%)	0/5 (0%)	0/15 (0%)	0/30 (0%)	1.000
Retinopathy of prematurity ≥ stage 2	3/50 (6%)	2/5 (40%)	1/15 (7%)	0/30 (0%)	0.008
PMA at stop of Invasive respiratory support, weeks	31.3 (30.6–32.7)	30.3 (29.2–31.2)	31.1 (30.6–31.4)	33.1 (32.7–34.6)	<0.001
PMA at stop of Non-invasive respiratory support, weeks	35.1 (34.4–36.1)	35.4 (34.4–36.3)	35.1 (34.3–36.3)	35.3 (34.9–36.0)	0.965
PMA at stop of supplemental O_2_, weeks	35.3 (34.6–36.0)	36.1 (35.2–36.7)	34.7 (31.9–35.7)	35.4 (34.9–36.0)	0.108
PMA at stop of caffeine therapy, weeks	35.9 (35.0–36.5)	36.0 (35.6–36.4)	35.4 (34.9–35.9)	36.3 (35.9–37.5)	0.118
Number of HRV measurements, days	879	171	401	307	—

Values are presented as median (interquartile range [IQR]) for continuous variables and n/N (%) for categorical variables. *p*-values represent overall comparisons among the three gestational-age groups. Continuous variables were compared using the Kruskal–Wallis test, and categorical variables were compared using the Fisher–Freeman–Halton exact test. *p*-values were not calculated for gestational age because it was used to define the groups or for the number of HRV measurements because it represents the number of repeated observations rather than an infant-level characteristic. Chorioamnionitis data were unavailable in one infant born at 28–31 weeks’ gestation and nine infants born at ≥32 weeks’ gestation because placental histopathological examination was not performed.

**Table 2:** Associations of postmenstrual age, body weight z-score, and day of life with heart rate variability indices determined using linear mixed-effects models.

	Postmenstrual Age		Body Weight z-Score		Day of Life	
HRV Index	β (95% CI)	*p* Value	β (95% CI)	*p* Value	β (95% CI)	*p*-Value
SDNNI (ms)	1.74 (1.17–2.32)	<0.001	−0.89 (−2.03–0.25)	0.126	−0.11 (−0.19–−0.02)	0.011
SDNN (ms)	1.21 (0.83–1.59)	<0.001	−0.74 (−1.48–0.00)	0.051	−0.08 (−0.14–−0.03)	0.004
RMSSD (ms)	0.68 (0.40–0.96)	<0.001	−0.37 (−0.93–0.19)	0.191	−0.05 (−0.09–−0.01)	0.015
VLF power (ms^2^)	3.10 (1.69–4.50)	<0.001	−0.07 (−2.98–2.83)	0.960	−0.25 (−0.45–−0.05)	0.015
LF power (ms^2^)	22.60 (14.85–30.35)	<0.001	−8.49 (−23.66–6.68)	0.272	−1.38 (−2.49–−0.26)	0.015
HF power (ms^2^)	3.89 (1.81–5.97)	<0.001	0.56 (−3.94–5.06)	0.806	−0.22 (−0.51–0.08)	0.151

Data are presented as regression coefficients (β) with 95% confidence intervals (CI). Linear mixed-effects models included postmenstrual age (PMA), body weight z-score, and day of life as fixed effects and infant ID as a random intercept to account for repeated measurements within infants. The regression coefficients represent the estimated change in each HRV index associated with a 1-week increase in PMA, a 1-unit increase in body weight z-score, or a 1-day increase in day of life, after adjustment for the other predictors. A two-sided *p*-value < 0.05 was considered statistically significant.

**Table 3:** Associations of postmenstrual age and clinical factors with heart rate variability indices in multivariable linear mixed-effects models.

	PMA, per Week		Invasive Respiratory Support		Non-Invasive Respiratory Support		Oxygen Supplementation		Catecholamine Use		Caffeine Exposure	
HRV Index	β (95% CI)	*p*-Value	β (95% CI)	*p*-Value	β (95% CI)	*p*-Value	β (95% CI)	*p*-Value	β (95% CI)	*p*-Value	β (95% CI)	*p*-Value
SDNNI (ms)	0.55 (0.37–0.73)	<0.001	−1.16 (−2.61–0.29)	0.118	−0.01 (−0.82–0.80)	0.987	−0.59 (−1.43–0.24)	0.167	−1.26 (−2.18–−0.34)	0.007	1.59 (0.30–2.87)	0.015
SDNN (ms)	0.38 (0.27–0.50)	<0.001	−1.23 (−2.14–−0.33)	0.008	−0.27 (−0.78–0.23)	0.289	−0.01 (−0.54–0.50)	0.952	−1.13 (−1.70–−0.55)	<0.001	0.03 (−0.76–0.84)	0.932
RMSSD (ms)	0.17 (0.09–0.25)	<0.001	−0.93 (−1.60–−0.26)	0.006	−0.36 (−0.73–0.01)	0.058	0.27 (−0.11–0.66)	0.165	−0.59 (−1.01–−0.16)	0.006	0.21 (−0.37–0.81)	0.476
VLF power (ms^2^)	0.40 (−0.01–0.82)	0.058	−3.06 (−6.45–0.31)	0.076	0.16 (−1.72–2.06)	0.861	0.10 (−1.84–2.06)	0.914	−2.55 (−4.69–−0.40)	0.020	1.26 (−1.73–4.26)	0.407
LF power (ms^2^)	4.82 (2.65–6.98)	<0.001	−16.58 (−34.01–0.84)	0.062	−7.53 (−17.30–2.22)	0.130	−3.24 (−13.30–6.81)	0.527	−15.43 (−26.47–−4.38)	0.006	1.79 (−13.63–17.23)	0.819
HF power (ms^2^)	0.76 (0.26–1.26)	0.003	−3.08 (−7.10–0.92)	0.132	−1.05 (−3.30–1.19)	0.357	0.84 (−1.47–3.16)	0.477	−1.75 (−4.29–0.79)	0.177	0.23 (−3.37–3.84)	0.898

Values are regression coefficients (β) with 95% confidence intervals (CI) obtained from multivariable linear mixed-effects models. Fixed effects included postmenstrual age (PMA), invasive respiratory support, non-invasive respiratory support, oxygen supplementation, catecholamine use, and caffeine exposure. A random intercept for each infant was included to account for repeated measurements within individuals. Caffeine exposure was defined as any previous or ongoing caffeine treatment, whereas infants with no history of caffeine therapy served as the reference group. Analyses were based on 879 observations from 50 infants. A two-sided *p*-value <0.05 was considered statistically significant.

## Data Availability

The original contributions presented in this study are included in the article and the Supplementary Materials. Further inquiries can be directed to the corresponding author.

## Supplementary Materials

The following supporting information can be downloaded at https://www.mdpi.com/article/10.3390/bios16100586/s1. Supplementary Figure S1: Individual longitudinal trajectories of time-domain heart rate variability according to gestational age. Spaghetti plots illustrating individual longitudinal trajectories of time-domain heart rate variability (HRV) indices stratified by gestational age (<28 weeks, 28–31 weeks, and ≥32 weeks). Each gray line represents repeated measurements from a single infant, and the solid black line represents the group mean. Panels show (**a**) Standard Deviation of the Normal-to-Normal interval Index (SDNNI), (**b**) Standard Deviation of the Normal-to-Normal interval (SDNN), and (**c**) root mean square of successive differences (RMSSD); Supplementary Figure S2: Individual longitudinal trajectories of frequency-domain heart rate variability according to gestational age. Spaghetti plots illustrating individual longitudinal trajectories of frequency-domain heart rate variability (HRV) indices stratified by gestational age (<28 weeks, 28–31 weeks, and ≥32 weeks). Each gray line represents repeated measurements from a single infant, and the solid black line represents the group mean. Panels show (**a**) very-low-frequency (VLF) power, (**b**) low-frequency (LF) power, and (**c**) high-frequency (HF) power; Supplementary Table S1. Infant-level coverage and distribution of analyzed 24 h HRV recordings across postmenstrual age weeks (26–36 weeks); Supplementary Table S2. Linear mixed-effects analysis of HRV indices between infants born at <32 and ≥32 weeks’ gestation, restricted to measurements obtained between 33 and 36 weeks’ postmenstrual age; Supplementary Table S3. Minimal dataset for longitudinal 24-h heart rate variability trajectories in preterm infants.

## Abbreviations

The following abbreviations are used in this manuscript:

ANS	autonomic nervous system
CI	confidence interval
CPAP	continuous positive airway pressure
DOL	day of life
ECG	electrocardiography
HF	high-frequency power
HFNC	high-flow nasal cannula
HRV	heart rate variability
IQR	interquartile range
LF	low-frequency power
NICU	neonatal intensive care unit
PMA	postmenstrual age
RRI	RR interval
RMSSD	root mean square of successive differences
SDNN	standard deviation of normal-to-normal intervals
SDNNI	mean of the standard deviations of normal-to-normal intervals for all 5 min segments
VLF	very-low-frequency power
