# Supplementary material for: Longitudinal Trajectories of Autonomic Maturation in Preterm Infants Using Serial 24-Hour Heart Rate Variability Monitoring
Source: Biosensors (Basel). 2026 Oct 19;16(10):586. doi: 10.3390/bios16100586 (PMC13702146; doi:10.3390/bios16100586)
Supplement: Supplementary file 1 [file biosensors-16-00586-s001.zip › biosensors-4546206-supplementary.pdf]

biosensors

MDPI

Supplementary Materials 1

1 Supplementary Figures 2

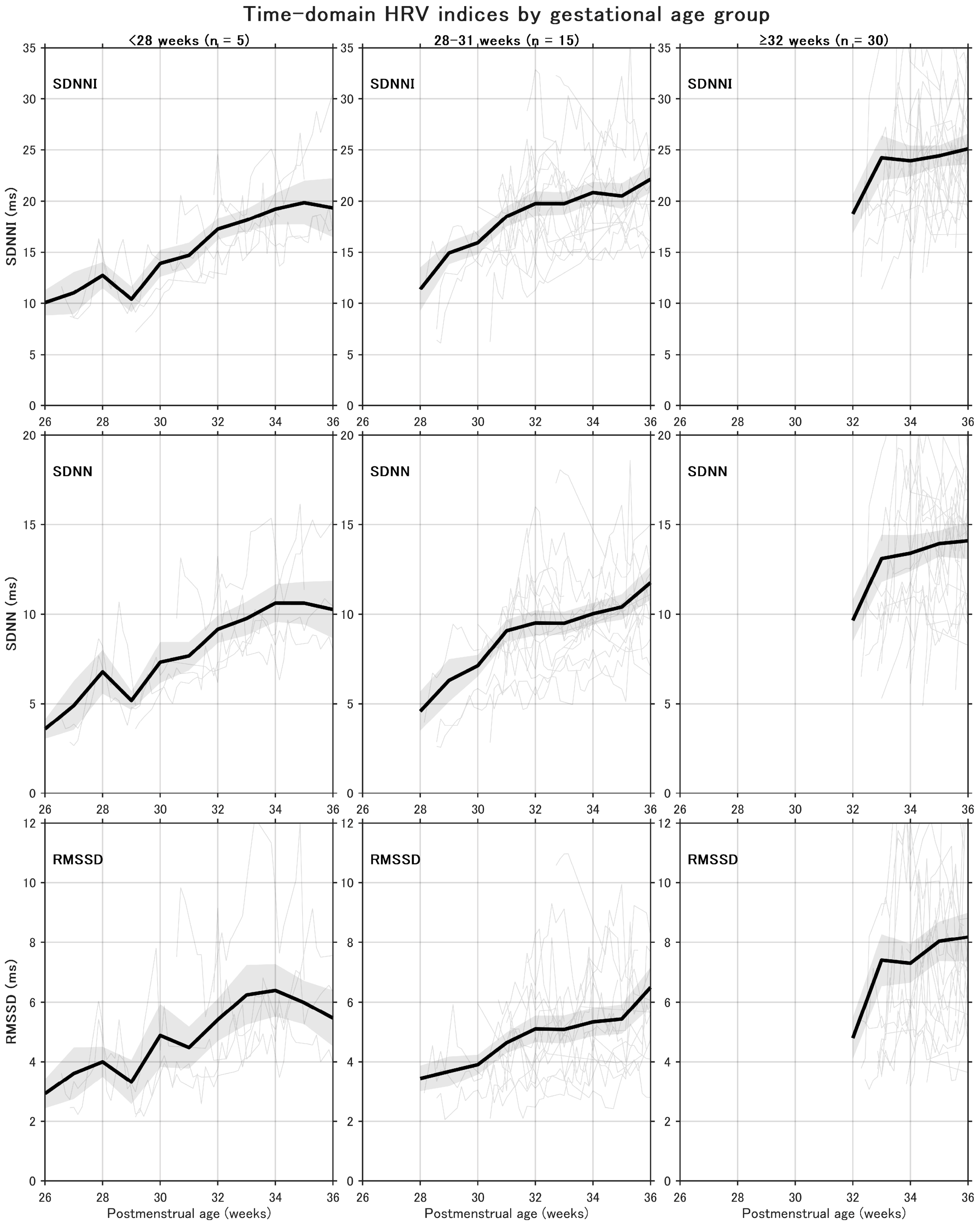

3

Supplementary Figure S1. Individual longitudinal trajectories of time-domain heart rate variability according to gestational age. Spaghetti plots illustrating individual longitudinal trajectories of time-domain 4 5

heart rate variability (HRV) indices stratified by gestational age (<28 weeks, 28–31 weeks, and ≥32 weeks). Each gray line represents repeated measurements from a single infant, and the solid black line represents the group mean. Panels show (a) Standard Deviation of the Normal-to-Normal interval Index (SDNNI), (b) Standard Deviation of the Normal-to-Normal interval (SDNN), and (c) root mean square of successive differences (RMSSD).

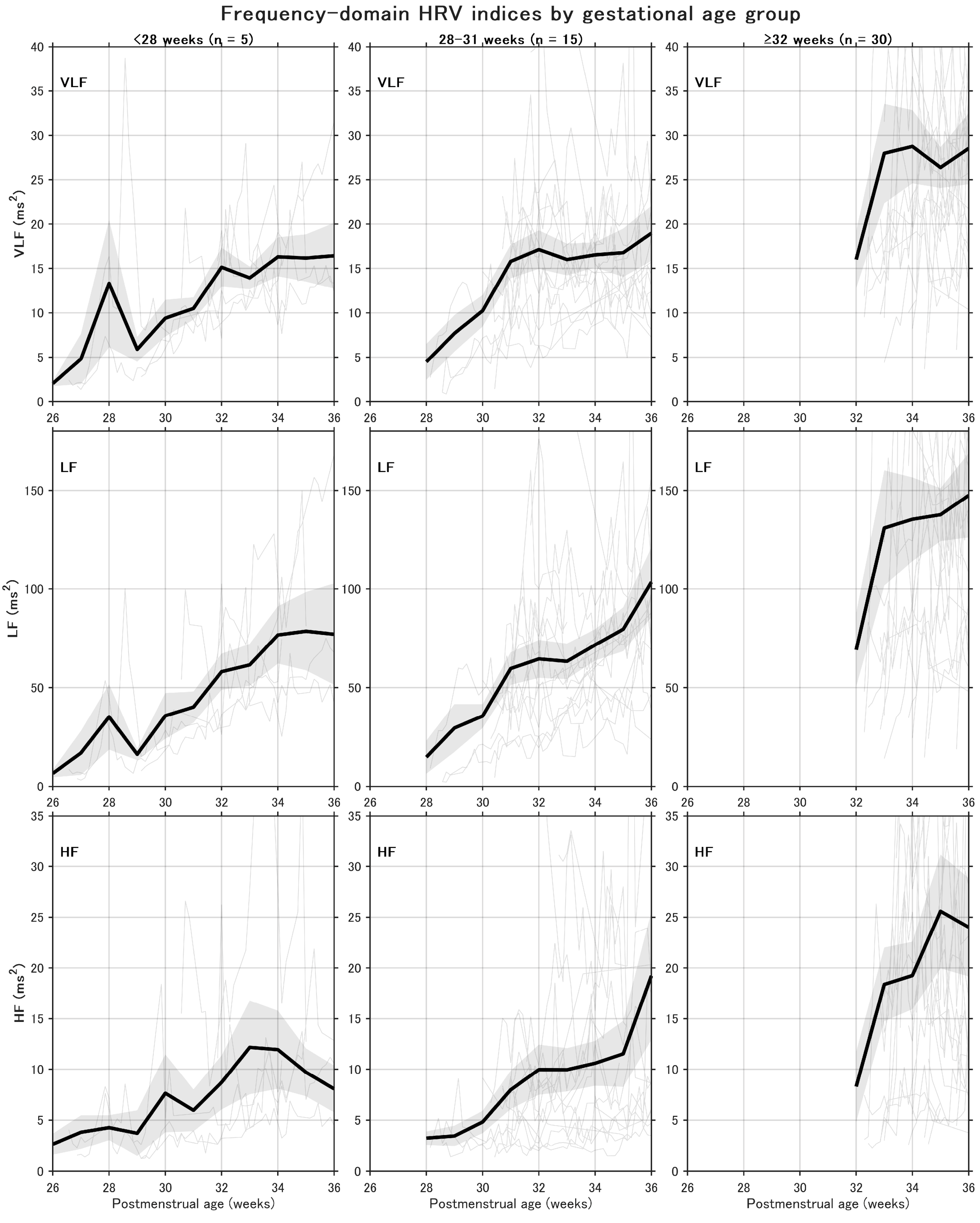

Supplementary Figure S2. Individual longitudinal trajectories of frequency-domain heart rate variability according to gestational age. Spaghetti plots illustrating individual longitudinal trajectories of frequency-

domain heart rate variability (HRV) indices stratified by gestational age (<28 weeks, 28–31 weeks, and ≥32 weeks). Each gray line represents repeated measurements from a single infant, and the solid black line represents the group mean. Panels show (a) very-low-frequency (VLF) power, (b) low-frequency (LF) power, and (c) high-frequency (HF) power.

2 Supplementary Tables

Supplementary Table S1. Infant-level coverage and distribution of analyzed 24-h HRV recordings across postmenstrual age weeks (26–36 weeks)

ID	GA group	Recordings	First PMA	Last PMA	PMA weeks covered	26 wk	27 wk	28 wk	29 wk	30 wk	31 wk	32 wk	33 wk	34 wk	35 wk	36 wk
1	<28 weeks	53	26.86	36.86	26, 27, 28, 29, 30, 31, 32, 33, 34, 35, 36	1	4	6	5	5	5	6	5	5	5	6
2	<28 weeks	12	31.86	34.14	31, 32, 33, 34	0	0	0	0	0	1	4	5	2	0	0
3	<28 weeks	50	26.57	36.86	26, 27, 28, 29, 30, 31, 32, 33, 34, 35, 36	3	5	5	5	3	3	5	5	5	6	5
4	<28 weeks	35	29.14	36.29	29, 30, 31, 32, 33, 34, 35, 36	0	0	0	5	5	5	5	4	5	3	3
5	<28 weeks	21	30.57	35.00	30, 31, 32, 33, 34, 35	0	0	0	0	3	4	4	4	5	1	0
6	28–31 weeks	16	30.43	33.00	30, 31, 32, 33	0	0	0	0	4	5	6	1	0	0	0
7	28–31 weeks	22	30.00	34.86	30, 31, 32, 33, 34	0	0	0	0	3	4	4	6	5	0	0
8	28–31 weeks	33	30.86	36.86	30, 31, 32, 33, 34, 35, 36	0	0	0	0	1	5	5	5	6	5	6
35	28–31 weeks	27	31.71	36.86	31, 32, 33, 34, 35, 36	0	0	0	0	0	2	5	6	5	4	5
10	28–31 weeks	41	28.57	36.71	28, 29, 30, 31, 32, 33, 34, 35, 36	0	0	3	4	5	5	7	5	4	4	4
11	28–31 weeks	24	30.71	35.57	30, 31, 32, 33, 34, 35	0	0	0	0	2	6	5	5	3	3	0
12	28–31 weeks	27	30.71	36.71	30, 31, 32, 33, 34, 35, 36	0	0	0	0	2	6	6	3	1	4	5
13	28–31 weeks	19	30.29	36.86	30, 31, 32, 33, 36	0	0	0	0	4	4	4	1	0	0	6
14	28–31 weeks	30	31.57	36.86	31, 32, 33, 34, 35, 36	0	0	0	0	0	3	5	6	5	5	6
15	28–31 weeks	18	33.00	36.14	33, 34, 35, 36	0	0	0	0	0	0	0	6	5	5	2
16	28–31 weeks	37	28.14	36.86	28, 29, 30, 31, 32, 34, 35, 36	0	0	5	5	6	6	3	0	1	5	6
17	28–31 weeks	38	28.57	36.71	28, 29, 30, 31, 32, 33, 34, 35, 36	0	0	3	4	6	5	6	5	5	2	2
18	28–31 weeks	29	31.00	36.57	31, 32, 33, 34, 35, 36	0	0	0	0	0	4	5	5	5	6	4
19	28–31 weeks	23	30.71	35.14	30, 31, 32, 33, 34, 35	0	0	0	0	2	5	5	4	5	2	0
20	28–31 weeks	17	32.71	36.71	32, 33, 34, 35, 36	0	0	0	0	0	0	2	2	3	5	5
21	≥32 weeks	6	35.57	36.86	35, 36	0	0	0	0	0	0	0	0	0	3	3
22	≥32 weeks	4	36.14	36.57	36	0	0	0	0	0	0	0	0	0	0	4
23	≥32 weeks	16	33.00	35.71	33, 34, 35	0	0	0	0	0	0	0	6	5	5	0
24	≥32 weeks	5	36.00	36.86	36	0	0	0	0	0	0	0	0	0	0	5

25	≥32 weeks	13	34.00	36.29	34, 35, 36	0	0	0	0	0	0	0	0	5	6	2
26	≥32 weeks	3	33.00	33.29	33	0	0	0	0	0	0	0	3	0	0	0
27	≥32 weeks	4	35.43	35.86	35	0	0	0	0	0	0	0	0	0	4	0
28	≥32 weeks	3	33.29	33.57	33	0	0	0	0	0	0	0	3	0	0	0
29	≥32 weeks	10	33.86	36.86	33, 34, 35, 36	0	0	0	0	0	0	0	1	5	2	2
30	≥32 weeks	15	32.86	35.71	32, 33, 34, 35	0	0	0	0	0	0	1	5	5	4	0
31	≥32 weeks	6	36.00	36.86	36	0	0	0	0	0	0	0	0	0	0	6
32	≥32 weeks	14	32.86	36.14	32, 33, 34, 35, 36	0	0	0	0	0	0	1	5	1	6	1
33	≥32 weeks	10	32.29	34.14	32, 33, 34	0	0	0	0	0	0	3	5	2	0	0
34	≥32 weeks	15	33.43	36.86	33, 34, 35, 36	0	0	0	0	0	0	0	4	4	4	3
35	≥32 weeks	15	32.43	36.86	32, 33, 34, 36	0	0	0	0	0	0	4	2	5	0	4
36	≥32 weeks	8	34.14	35.71	34, 35	0	0	0	0	0	0	0	0	4	4	0
37	≥32 weeks	8	33.86	35.00	33, 34, 35	0	0	0	0	0	0	0	1	6	1	0
38	≥32 weeks	5	35.71	36.29	35, 36	0	0	0	0	0	0	0	0	0	2	3
39	≥32 weeks	26	32.29	36.71	32, 33, 34, 35, 36	0	0	0	0	0	0	4	6	5	6	5
40	≥32 weeks	7	34.86	36.86	34, 35, 36	0	0	0	0	0	0	0	0	1	2	4
41	≥32 weeks	10	34.86	36.29	34, 35, 36	0	0	0	0	0	0	0	0	1	6	3
42	≥32 weeks	19	33.57	36.71	33, 34, 35, 36	0	0	0	0	0	0	0	3	5	6	5
43	≥32 weeks	19	33.00	36.86	33, 34, 35, 36	0	0	0	0	0	0	0	5	4	6	4
44	≥32 weeks	7	35.71	36.86	35, 36	0	0	0	0	0	0	0	0	0	2	5
45	≥32 weeks	7	35.71	36.71	35, 36	0	0	0	0	0	0	0	0	0	2	5
46	≥32 weeks	25	32.43	36.86	32, 33, 34, 35, 36	0	0	0	0	0	0	3	5	5	6	6
47	≥32 weeks	3	35.57	35.86	35	0	0	0	0	0	0	0	0	0	3	0
48	≥32 weeks	4	34.57	35.00	34, 35	0	0	0	0	0	0	0	0	3	1	0
49	≥32 weeks	8	34.57	36.29	34, 35, 36	0	0	0	0	0	0	0	0	2	4	2
50	≥32 weeks	12	34.43	36.86	34, 35, 36	0	0	0	0	0	0	0	0	4	3	5
Subtotal	<28 weeks	171	-	-	-	4	9	11	15	16	18	24	23	22	15	14
Subtotal	28–31 weeks	401	-	-	-	0	0	11	13	35	60	68	60	53	50	51
Subtotal	≥32 weeks	307	-	-	-	0	0	0	0	0	0	16	54	72	88	77
Overall	All	879	-	-	-	4	9	22	28	51	78	108	137	147	153	142

Each row represents one infant. "GA group" indicates gestational age at birth (<28, 28–31, or ≥32 weeks).
"Recordings" indicates the total number of analyzed 24-h HRV recordings included in the final analyses.

"First PMA" and "Last PMA" indicate the PMA at the first and last analyzed recordings, respectively.
"PMA weeks covered" indicates the postmenstrual age weeks during which at least one analyzed recording was available.
Columns labeled 26–36 wk show the number of analyzed recordings obtained during each PMA week.
Subtotal rows summarize recordings within each gestational-age group, and the overall row summarizes recordings across all infants.

biosensors

MDPI

Supplementary Table S2. Linear mixed-effects analysis of HRV indices between infants born at <32 and ≥32 weeks' gestation, restricted to measurements obtained between 33 and 36 weeks' postmenstrual age.

HRV_index	β (95% CI)	P value
SDNNI, ms	-4.491 (-7.369 to -1.613)	0.002
SDNN, ms	-3.483 (-5.325 to -1.642)	<0.001
RMSSD, ms	-2.461 (-3.820 to -1.101)	<0.001
VLF power, ms^2	-13.600 (-20.472 to -6.727)	<0.001
LF power, ms^2	-63.069 (-99.675 to -26.463)	<0.001
HF power, ms^2	-9.966 (-20.240 to 0.307)	0.057

Linear mixed-effects models were restricted to measurements obtained between 33 and 36 weeks' postmenstrual age (PMA).
The models included PMA and gestational age group (<32 vs ≥32 weeks) as fixed effects and infant ID as a random intercept to account for repeated measurements.
The ≥32-week group served as the reference category; therefore, negative β coefficients indicate lower HRV values in infants born at <32 weeks than in those born at ≥32 weeks.

Supplementary Table S3. Minimal dataset for longitudinal 24-hour heart rate variability trajectories in preterm infants

Study_ID	GA_group	PMA_weeks	SDNNI_ms	SDNN_ms	RMSSD_ms	VLF_power_ms²	LF_power_ms²	HF_power_ms²
P001	28-31	30.43	6.2452	2.8247	2.0842	1.4154	4.5136	1.3522
P001	28-31	30.57	13.0546	5.8518	3.2174	6.0888	22.4296	2.6966
P001	28-31	30.71	11.0721	6.1086	3.2086	5.4968	25.1724	2.6884
P001	28-31	30.86	10.3933	5.4861	2.9723	4.758	19.6971	2.5025
P001	28-31	31.14	16.6101	9.4287	4.9803	10.9113	68.1353	14.6753
P001	28-31	31.29	18.6644	10.7118	5.0835	15.211	89.5761	15.8845
P001	28-31	31.43	19.7635	11.2828	5.1013	21.9176	96.1862	15.3894
P001	28-31	31.57	20.5501	10.4379	4.5624	20.1521	80.2532	12.4543
P001	28-31	31.86	21.0602	9.8937	4.4738	17.402	66.6001	4.9023
P001	28-31	32	21.4641	10.0695	4.8157	15.0537	68.238	5.694
P001	28-31	32.14	18.9631	9.3736	4.4171	12.5289	59.9715	4.7909
P001	28-31	32.29	21.7256	10.4586	5.1789	14.4868	74.276	6.6943
P001	28-31	32.57	21.595	10.6152	5.3219	12.862	76.2988	7.073
P001	28-31	32.71	23.2473	11.5501	5.8886	15.9434	92.4283	9.1341
P001	28-31	32.86	20.7384	10.9692	6.2044	14.8508	79.4175	10.5861
P001	28-31	33	22.7385	11.0089	5.3441	15.785	83.3107	7.8477
P002	>=32	35.57	17.9279	5.7815	4.3316	5.865	18.8275	4.4483
P002	>=32	35.71	20.8766	9.9211	5.9616	16.0492	63.4774	8.6174
P002	>=32	35.86	29.663	16.3857	11.0185	40.4724	167.5036	28.4322
P002	>=32	36.29	47.2012	25.9961	21.0348	70.2788	403.4809	118.0039
P002	>=32	36.43	21.5466	13.4622	10.9353	21.2283	90.5581	36.0066
P002	>=32	36.86	35.0933	18.4013	12.4242	38.4652	221.396	45.3669
P003	>=32	36.14	20.2007	10.4938	7.3449	17.8266	63.9155	15.5925
P003	>=32	36.29	16.6917	9.964	7.0463	10.5472	60.5813	14.2049
P003	>=32	36.43	18.305	10.9572	9.5864	10.4424	60.728	28.0023
P003	>=32	36.57	14.086	7.8081	4.5825	7.3469	41.1443	6.4769
P004	>=32	33	14.107	7.2128	3.4987	9.4455	36.0092	3.002
P004	>=32	33.14	20.2061	11.4421	5.2463	21.345	94.0621	6.3961
P004	>=32	33.43	19.1867	10.6963	5.3246	18.9471	78.5073	6.5588
P004	>=32	33.57	17.8245	10.3178	5.1182	15.0263	75.766	6.3575
P004	>=32	33.71	19.7389	11.2733	6.4412	17.2743	85.6419	10.2324
P004	>=32	33.86	18.0664	10.5137	5.1755	17.1918	76.9739	6.5158
P004	>=32	34.14	20.7793	12.7606	6.65	21.3361	112.1661	11.0382

P004	>=32	34.29	21.6042	12.9946	7.5365	20.7235	116.1986	14.879
P004	>=32	34.43	22.9546	13.8833	7.1518	22.9366	137.5161	13.1657
P004	>=32	34.57	23.2674	14.753	8.1577	22.6652	150.2733	17.3499
P004	>=32	34.71	22.8261	14.2484	8.3465	22.7825	136.2007	18.843
P004	>=32	35	23.4362	13.3168	7.2834	21.7146	120.6961	14.6944
P004	>=32	35.14	22.0058	12.7738	6.5726	20.6485	113.0997	11.1385
P004	>=32	35.29	24.1825	15.1788	8.3151	23.9482	152.6305	18.284
P004	>=32	35.57	21.8532	13.0416	7.1747	19.9276	117.1906	13.6756
P004	>=32	35.71	22.925	12.9366	6.8935	20.922	115.715	12.4375
P005	28-31	30.71	18.3449	8.0992	4.7286	11.9026	38.6193	6.0692
P005	28-31	30.86	19.2676	8.9209	5.396	14.3638	48.1552	7.4554
P005	28-31	30	19.4673	9.4388	5.9502	14.6531	53.7087	9.1362
P005	28-31	31.14	21.663	10.4989	6.0453	18.7051	67.9012	9.086
P005	28-31	31.29	20.1942	11.0525	6.6822	20.3097	71.6682	11.7324
P005	28-31	31.71	22.4601	10.8789	5.7052	21.0827	74.1531	7.7608
P005	28-31	31.86	25.4194	12.1743	6.4421	24.2185	93.3849	9.9797
P005	28-31	32	23.2546	11.183	5.7198	21.9663	79.7182	8.242
P005	28-31	32.14	22.7032	10.9947	5.8959	19.4327	76.0574	8.7739
P005	28-31	32.29	24.7685	11.6134	6.1187	23.4934	85.0824	9.2055
P005	28-31	32.86	22.8233	10.104	5.0887	17.4749	65.5339	6.6161
P005	28-31	33	21.7881	9.7524	4.8786	15.5758	63.9478	6.276
P005	28-31	33.14	22.1677	9.6435	4.8553	15.9711	62.0775	6.3851
P005	28-31	33.29	21.3223	9.1787	4.5532	14.3284	55.8289	5.677
P005	28-31	33.43	20.9521	9.0102	4.9608	13.3319	52.0912	6.8014
P005	28-31	33.71	21.9315	9.6235	5.6711	13.5779	58.1666	9.2102
P005	28-31	33.86	21.5754	9.1306	5.6505	13.1753	53.3531	9.2551
P005	28-31	34	21.3341	10.3911	6.6486	13.9596	68.4472	12.4588
P005	28-31	34.14	23.4802	10.9155	6.3543	15.6045	78.3844	10.7166
P005	28-31	34.57	16.7534	8.9469	5.0066	10.6023	55.0255	6.0371
P005	28-31	34.71	22.1459	11.6091	6.1744	19.9932	97.1241	8.5846
P005	28-31	34.86	26.6618	13.4103	6.7482	23.5772	129.5825	10.6329
P006	>=32	36	19.3589	9.48	4.6671	15.0663	63.3296	5.0069
P006	>=32	36.14	17.7026	9.0555	4.6954	13.4441	56.8742	5.4849
P006	>=32	36.57	23.382	10.8133	5.7984	18.2593	80.8374	9.1709
P006	>=32	36.71	16.9391	8.3841	4.4537	10.0253	47.6532	5.58
P006	>=32	36.86	15.8321	8.0651	4.2652	8.7929	43.9824	5.2234
P007	28-31	30.86	18.5454	9.0664	4.2615	15.1512	56.5264	4.6484

P007	28-31	31	15.0802	6.5545	3.0162	8.4796	28.5427	2.4041
P007	28-31	31.14	17.5953	10.0164	5.292	15.403	67.6563	7.1445
P007	28-31	31.29	19.1591	11.0149	5.6267	19.1989	85.0492	8.5208
P007	28-31	31.71	26.0875	14.8072	6.5541	34.6052	160.3914	10.4981
P007	28-31	31.86	22.033	11.8758	5.0171	23.6011	102.6196	6.0586
P007	28-31	32	22.808	11.7677	4.9814	23.5628	100.3353	5.7674
P007	28-31	32.14	24.2771	12.3172	5.7017	24.8198	107.2442	7.8754
P007	28-31	32.57	25.4442	12.6232	5.7301	27.2178	109.4718	8.5202
P007	28-31	32.71	25.9502	12.3845	5.8214	23.9978	104.8754	9.292
P007	28-31	32.86	21.5327	10.028	4.5048	16.6512	69.284	6.0198
P007	28-31	33	18.747	8.569	3.3957	12.9297	52.5276	3.4091
P007	28-31	33.29	21.0569	10.1979	4.6901	15.9894	70.7266	6.2923
P007	28-31	33.43	19.9268	9.1179	3.6162	13.792	60.2046	3.4798
P007	28-31	33.57	20.5251	9.183	4.1343	13.4916	60.1248	4.8438
P007	28-31	33.71	22.1448	10.2033	4.9665	15.044	71.6989	6.6663
P007	28-31	34	22.7144	10.2678	5.0516	14.4389	75.0878	6.6819
P007	28-31	34.14	22.0503	10.5088	5.1911	15.2231	79.2868	7.1599
P007	28-31	34.29	22.155	10.7225	5.0553	15.7253	81.6224	6.7427
P007	28-31	34.43	21.2446	10.4407	4.9504	15.6907	77.2981	7.0422
P007	28-31	34.71	22.3587	11.3364	5.6209	17.6813	90.3316	8.6818
P007	28-31	34.86	20.2699	9.962	4.6822	12.6255	71.3786	5.962
P007	28-31	35	21.7657	10.2809	4.7967	14.3403	76.7696	6.2104
P007	28-31	35.14	18.3723	9.3216	4.0033	11.7134	64.6543	4.1726
P007	28-31	35.29	22.3923	10.7903	5.2642	14.1043	82.6817	7.613
P007	28-31	35.71	19.2361	9.3798	4.5781	11.3714	61.604	5.6994
P007	28-31	35.86	20.2057	11.4059	5.9266	15.3452	94.2008	9.2926
P007	28-31	36	21.5734	10.3455	5.561	14.3223	73.4297	8.4215
P007	28-31	36.14	20.3836	9.2765	4.394	11.6987	60.6561	4.8792
P007	28-31	36.43	23.5699	11.0101	5.5659	14.1998	83.6345	8.4629
P007	28-31	36.57	24.915	12.9854	6.8521	18.6428	117.8477	12.5855
P007	28-31	36.71	22.6711	11.8955	6.9802	14.4894	92.2619	14.3583
P007	28-31	36.86	22.6019	11.475	5.9246	14.8384	90.5432	9.6358
P008	>=32	34	12.4531	6.1257	3.1771	8.2289	24.4787	5.581
P008	>=32	34.14	17.1899	8.1078	3.6241	11.2958	47.2213	9.8891
P008	>=32	34.29	29.4517	16.3669	8.099	42.7182	195.1111	45.4608
P008	>=32	34.43	23.4119	13.6429	5.9966	31.8358	142.6025	27.966
P008	>=32	34.86	21.7271	11.6945	5.4018	19.1432	101.7544	22.7589

P008	>=32	35	20.9103	11.6322	5.3406	17.9855	101.3668	23.1849
P008	>=32	35.14	27.8387	14.6092	6.3472	30.4911	161.6177	33.038
P008	>=32	35.29	26.9071	15.2661	6.7931	29.1087	175.4651	39.6491
P008	>=32	35.57	27.4711	15.7148	7.2646	29.1847	184.6176	45.2358
P008	>=32	35.71	29.6325	17.5389	8.3784	32.8351	240.7199	60.2944
P008	>=32	35.86	28.1336	16.3659	7.2484	30.0652	211.0125	47.8022
P008	>=32	36	26.8919	16.4436	7.6263	31.0911	206.0155	50.7137
P008	>=32	36.29	28.1319	16.4408	7.5956	31.6662	205.9435	49.3277
P009	28-31	31.71	28.8136	13.9936	7.307	39.393	132.2386	39.0029
P009	28-31	31.86	30.5273	15.0859	7.494	39.7763	157.7846	43.7663
P009	28-31	32	32.867	15.9878	8.2612	45.6489	176.0975	53.2131
P009	28-31	32.14	32.4715	15.7072	8.4606	42.7057	164.8053	53.9416
P009	28-31	32.29	26.2499	13.0916	6.5967	32.7408	112.6018	34.4917
P009	28-31	32.71	25.7894	11.1254	5.1183	23.9072	82.5189	21.6553
P009	28-31	32.86	24.5895	10.5931	4.8302	20.8244	77.4752	18.6312
P009	28-31	33	27.0807	12.8886	6.1708	28.6616	112.1347	30.9452
P009	28-31	33.14	30.3364	13.261	6.5177	30.8596	121.1461	33.1581
P009	28-31	33.43	26.9989	11.8039	5.0955	25.5308	95.9581	21.7099
P009	28-31	33.57	29.262	11.8458	5.7505	23.3203	98.1028	25.9632
P009	28-31	33.71	24.8768	10.8551	4.7652	19.8883	84.5673	18.3826
P009	28-31	33.86	25.7666	11.225	5.0139	21.9659	87.9748	20.6736
P009	28-31	34	23.294	10.0058	4.372	16.8378	70.1017	15.7563
P009	28-31	34.29	26.9008	12.6892	6.3257	24.2995	109.3696	31.5332
P009	28-31	34.43	25.3354	11.397	5.2541	20.8547	91.7767	21.8866
P009	28-31	34.57	28.0089	12.3856	5.6242	23.1812	109.4369	29.6938
P009	28-31	34.71	25.3729	10.5507	5.0282	16.1999	78.7246	20.4403
P009	28-31	35.14	24.988	10.4432	5.0474	16.9416	75.5259	20.1681
P009	28-31	35.29	35.759	18.5986	8.6954	69.3514	247.1667	72.5121
P009	28-31	35.43	23.9033	11.1828	5.5803	17.6232	88.6383	24.0269
P009	28-31	35.86	26.7552	13.8637	6.7711	29.3676	141.8258	39.5538
P009	28-31	36	23.1949	12.5868	7.1403	21.2141	108.8508	39.7357
P009	28-31	36.14	22.1859	12.5725	7.7327	18.0098	102.5446	43.5104
P009	28-31	36.57	33.4258	19.8886	11.0941	65.9325	296.9399	102.633
P009	28-31	36.71	34.9878	19.5475	10.1422	44.228	288.9011	83.3662
P009	28-31	36.86	32.2666	18.5876	10.9401	47.6712	231.3082	90.5124
P010	>=32	33	26.0243	14.0799	8.8996	31.5521	123.8278	17.2006
P010	>=32	33.14	27.2767	15.9735	9.5143	39.8353	166.5444	19.8305

P010	>=32	33.29	23.1833	14.6716	8.7798	35.2253	140.2161	17.1966
P011	28-31	28.57	7.4807	2.8553	3.7718	1.0365	2.2181	3.2088
P011	28-31	28.71	11.7317	4.1629	4.3551	5.0618	7.3577	4.9074
P011	28-31	28.86	12.9928	3.777	3.6965	2.6885	5.8231	3.8734
P011	28-31	29.29	12.8342	3.7837	3.1232	3.1602	7.0967	2.2957
P011	28-31	29.57	13.9687	4.8146	3.668	5.017	12.8391	3.3172
P011	28-31	29.71	15.4274	5.4768	3.7039	7.1514	18.9516	3.0509
P011	28-31	29.86	17.1194	4.8189	3.3516	6.7922	12.1125	2.5548
P011	28-31	30.14	16.2288	4.6399	3.4517	4.7418	12.6129	2.821
P011	28-31	30.29	16.052	4.8481	4.1071	4.2671	11.7047	4.1764
P011	28-31	30.43	17.0926	5.547	3.3702	7.5871	18.2383	2.8159
P011	28-31	30.57	15.1396	4.7108	2.615	5.5316	13.4198	1.6893
P011	28-31	30.71	15.1288	5.4757	3.0339	7.4944	20.8254	2.1509
P011	28-31	31.14	15.2256	6.0407	3.4533	11.0443	23.669	2.7316
P011	28-31	31.29	15.3702	5.402	3.143	8.1541	19.8805	2.3862
P011	28-31	31.43	17.2783	6.2531	3.3971	10.4384	25.015	2.4973
P011	28-31	31.71	15.9669	6.2623	2.9978	12.0705	26.2651	1.9842
P011	28-31	31.86	17.1611	5.757	3.2688	8.9939	22.2553	2.5807
P011	28-31	32	15.8616	5.4792	3.1093	8.0691	19.5982	2.2292
P011	28-31	32.14	16.971	5.9221	3.2655	8.7453	23.5192	2.4246
P011	28-31	32.29	17.8449	6.5004	3.485	11.6594	27.5535	2.7657
P011	28-31	32.43	19.0226	6.3935	3.3209	10.4206	25.2524	2.4556
P011	28-31	32.57	19.551	6.5135	3.536	12.0459	29.888	2.6762
P011	28-31	32.71	16.9313	6.4879	3.4774	12.0692	29.6852	2.7743
P011	28-31	32.86	16.3059	5.1619	3.411	6.0415	15.5575	2.8169
P011	28-31	33.29	14.6519	4.9569	3.2966	5.9736	15.0829	2.6572
P011	28-31	33.43	18.7052	6.8888	4.5077	8.1617	31.4374	5.3917
P011	28-31	33.57	15.94	5.7909	3.7612	7.9609	19.367	3.4249
P011	28-31	33.71	17.145	5.6943	3.6533	7.5191	20.274	3.1983
P011	28-31	33.86	18.3678	6.2795	4.293	8.4355	22.5192	4.4539
P011	28-31	34.29	20.8815	6.7003	3.8689	9.1514	29.6027	3.8021
P011	28-31	34.43	20.8629	7.1387	3.8757	13.9094	34.7832	3.7278
P011	28-31	34.57	19.4865	6.5891	3.7424	8.9787	29.9627	3.5069
P011	28-31	34.71	19.6299	6.0882	3.446	8.3082	23.5424	2.9475
P011	28-31	35	17.3887	5.8267	3.5932	7.1061	21.4128	3.2739
P011	28-31	35.14	16.4684	5.1137	3.422	4.9682	16.2185	3.0862
P011	28-31	35.29	17.1795	8.7811	6.8145	9.9356	43.6792	14.8026

P011	28-31	35.43	17.4085	7.2302	6.281	9.4502	30.2151	9.1732
P011	28-31	36.29	15.309	6.2461	5.9508	6.1127	20.3166	8.3592
P011	28-31	36.43	17.687	7.6809	7.7709	6.4715	32.7877	14.0933
P011	28-31	36.57	17.2234	7.7436	7.8452	7.0062	29.16	15.0087
P011	28-31	36.71	17.1814	7.6734	9.2983	6.3673	23.6998	18.6752
P012	<28	26.86	8.7239	2.8536	2.4523	1.7874	3.9955	1.5499
P012	<28	27	8.6354	2.6588	2.4677	1.362	3.202	1.6425
P012	<28	27.14	9.1299	2.9538	2.225	2.1062	4.334	1.3784
P012	<28	27.29	10.017	4.0465	2.4863	4.0245	9.6188	1.6433
P012	<28	27.86	16.0715	8.6462	6.0443	15.1477	53.7228	8.2982
P012	<28	28	14.747	8.2282	5.2142	16.3569	44.5663	7.1639
P012	<28	28.14	12.3336	6.5594	3.0162	10.7109	31.4051	2.3023
P012	<28	28.29	11.4159	6.0241	2.6921	10.4278	27.4714	1.6627
P012	<28	28.57	15.1694	10.6842	4.2387	38.7523	100.2469	3.8538
P012	<28	28.71	16.2664	9.0474	4.0818	28.7022	63.4978	3.5087
P012	<28	28.86	13.9446	8.3225	3.7757	22.919	49.7094	2.868
P012	<28	29.14	10.3674	4.8863	2.2412	8.5225	15.0613	1.2321
P012	<28	29.29	10.1015	5.0259	2.3063	8.0422	16.4424	1.2994
P012	<28	29.43	13.3201	6.1569	3.0569	9.4505	27.1208	2.1252
P012	<28	29.57	15.7691	6.6554	3.3978	11.6412	27.4134	3.0006
P012	<28	29.71	14.7928	5.5514	2.8236	8.7744	18.1843	2.035
P012	<28	30.14	13.9019	5.9261	3.9576	7.6783	21.0414	3.5801
P012	<28	30.29	14.3933	5.339	3.7194	5.3964	16.8007	3.333
P012	<28	30.43	13.2324	5.0121	3.1214	6.0284	16.2393	2.2958
P012	<28	30.57	13.372	5.9079	4.0086	6.8764	21.4945	3.6129
P012	<28	30.71	14.0794	6.2174	3.7216	9.0007	24.9253	3.1998
P012	<28	31	12.9223	6.1355	3.9387	7.6964	24.0902	3.9637
P012	<28	31.14	12.2806	6.3455	3.4926	10.1253	29.8046	2.9156
P012	<28	31.29	12.1431	6.4802	3.5555	8.8938	30.6483	2.9012
P012	<28	31.43	12.9527	6.3795	3.4902	10.1965	29.7724	2.8706
P012	<28	31.86	12.4611	7.0023	3.5034	10.1976	39.2633	2.6435
P012	<28	32	16.6819	6.1765	3.7625	7.1363	26.1434	3.4299
P012	<28	32.14	16.0243	8.9813	4.6567	21.6962	61.012	4.7016
P012	<28	32.29	15.6589	7.7456	4.0857	15.233	44.4702	3.9558
P012	<28	32.57	15.6655	9.1479	4.7288	22.4571	67.0788	5.3307
P012	<28	32.71	17.1202	10.0308	5.5356	23.6748	81.1483	7.0108
P012	<28	32.86	20.5778	11.0726	5.9275	29.1221	100.903	7.6345

P012	<28	33	14.6996	7.69	4.0896	16.005	40.8046	4.2461
P012	<28	33.43	15.9302	7.8735	4.5736	14.5537	42.856	5.0145
P012	<28	33.57	21.8023	7.6483	4.9415	11.111	37.5073	5.6335
P012	<28	33.71	14.8547	7.1619	4.2516	9.9691	32.7338	4.5259
P012	<28	33.86	18.2203	8.3709	4.9244	15.0817	44.7727	6.168
P012	<28	34.14	13.6419	6.3174	3.4769	7.6436	25.2001	3.2425
P012	<28	34.29	14.0015	7.419	4.008	11.6577	38.522	3.6606
P012	<28	34.43	17.9685	9.6956	5.4618	21.6413	67.5012	6.8822
P012	<28	34.57	16.6555	8.7649	4.8641	16.2501	50.4089	5.9753
P012	<28	34.71	16.3491	8.3034	4.6486	13.5901	46.8882	5.3627
P012	<28	35.14	16.5545	8.4023	4.5941	14.1176	47.6763	5.2034
P012	<28	35.29	18.5356	8.8475	4.5598	15.3288	54.3322	5.4178
P012	<28	35.43	18.6049	8.8002	4.8975	13.2222	52.4788	6.6647
P012	<28	35.57	16.1483	8.0553	4.0006	13.4053	42.6241	4.357
P012	<28	35.86	19.4792	8.7481	4.4083	14.1679	51.5858	5.2919
P012	<28	36	16.8753	8.0118	3.9739	12.2479	41.9162	4.4526
P012	<28	36.14	15.0653	7.1426	3.3522	10.9051	33.16	2.9992
P012	<28	36.29	15.3829	7.05	3.4075	10.478	31.85	3.0998
P012	<28	36.43	16.6547	7.5266	3.9094	10.2503	35.6621	4.1898
P012	<28	36.71	15.5774	7.4528	3.4435	14.2926	39.7037	2.9276
P012	<28	36.86	14.7994	6.6535	3.0994	9.8216	28.7084	2.493
P013	28-31	30.71	20.5661	9.4014	6.0899	17.1771	53.4332	8.108
P013	28-31	30.86	22.6546	10.3114	5.5776	21.4713	66.2138	6.8108
P013	28-31	31	20.9362	9.6947	6.0374	17.7949	54.7439	9.3156
P013	28-31	31.14	21.2541	9.5026	5.3339	17.7776	56.7362	6.9072
P013	28-31	31.29	17.7999	7.9319	4.367	13.8747	37.5698	5.0542
P013	28-31	31.57	19.9327	9.1742	4.9383	15.7343	53.1789	6.2399
P013	28-31	31.71	20.4557	9.9798	5.7141	17.7084	60.5733	8.2209
P013	28-31	31.86	24.4338	11.915	6.6447	24.7441	89.6342	10.3742
P013	28-31	32	21.3038	10.8657	6.0881	21.4187	72.3719	9.3954
P013	28-31	32.14	17.5804	9.986	6.3564	16.0672	59.2304	11.6379
P013	28-31	32.43	14.6616	7.9958	5.0282	10.991	38.7819	7.1258
P013	28-31	32.57	14.1647	7.0585	4.5838	10.9859	28.0885	5.7595
P013	28-31	32.71	15.9255	7.7966	4.8982	12.8759	35.2341	6.3782
P013	28-31	33.29	17.0488	8.5862	5.4429	14.204	42.234	8.4154
P013	28-31	33.43	16.9465	8.4485	5.7748	12.8708	39.5054	9.6665
P013	28-31	33.57	19.8152	9.6881	6.6226	17.9353	51.9265	11.8568

P013	28-31	33.71	17.1908	8.5343	5.9637	12.7566	40.6485	9.9778
P013	28-31	33.86	17.1513	10.0063	7.4606	14.5983	54.862	15.9236
P013	28-31	34.57	17.5271	8.4634	4.8444	14.5323	42.9452	6.491
P013	28-31	34.71	18.8133	8.9435	5.1708	15.2867	47.3987	7.0006
P013	28-31	34.86	18.6436	8.9384	4.9096	14.4654	49.5109	6.4373
P013	28-31	35.14	16.8447	8.3973	5.9965	11.5791	39.445	9.9917
P013	28-31	35.43	19.2491	10.0995	7.736	14.8686	55.8381	16.2189
P013	28-31	35.57	21.0601	11.1203	7.7324	19.3756	69.7918	15.7231
P014	28-31	30.71	21.154	7.502	4.1474	13.4732	36.6642	3.4758
P014	28-31	30.86	21.8047	8.5446	4.562	15.2407	48.7107	4.3166
P014	28-31	31	19.7755	8.4524	4.3808	15.2044	47.4836	4.1258
P014	28-31	31.14	17.9056	7.7049	4.1748	13.3955	37.4251	4.0051
P014	28-31	31.29	18.4649	8.1585	5.036	15.5917	41.0164	5.9503
P014	28-31	31.57	20.875	9.4779	4.6067	20.1676	58.7862	4.8882
P014	28-31	31.71	17.7357	8.042	3.9182	15.7033	42.4147	3.6799
P014	28-31	31.86	16.0504	7.4186	3.3274	13.4632	36.1377	2.8592
P014	28-31	32	13.8771	6.9225	3.2643	12.1554	29.766	2.7625
P014	28-31	32.14	18.3239	8.3513	3.973	14.9958	44.4336	3.967
P014	28-31	32.43	16.6636	8.7412	4.7378	15.002	46.6356	6.0137
P014	28-31	32.57	19.5686	10.5637	8.1659	18.008	59.3445	18.547
P014	28-31	32.71	19.9611	9.5796	6.5256	19.1416	54.6491	10.2469
P014	28-31	32.86	19.5654	9.1654	4.6962	17.3165	51.4962	5.5907
P014	28-31	33.57	20.1918	10.275	5.9111	19.0128	65.4568	9.1612
P014	28-31	33.71	19.3362	10.0771	6.1839	16.6647	58.9561	10.519
P014	28-31	33.86	19.4132	10.8479	7.8461	17.688	64.9251	17.4785
P014	28-31	34	17.795	9.1555	4.8546	15.4961	51.7679	6.2974
P014	28-31	35.14	17.9205	7.8988	3.8747	11.496	40.3493	4.0784
P014	28-31	35.29	17.131	7.8953	4.4465	10.2507	38.4426	5.6134
P014	28-31	35.71	17.6374	8.0761	5.2259	9.3716	38.3957	7.7679
P014	28-31	35.86	19.6633	8.2806	4.3992	12.1085	42.8158	5.1097
P014	28-31	36.14	19.7739	9.004	5.113	12.6783	51.3314	6.8531
P014	28-31	36.29	19.6832	9.1413	5.401	12.8832	51.5379	7.8674
P014	28-31	36.43	17.6951	8.3038	4.7693	10.1488	43.251	6.0951
P014	28-31	36.57	18.6649	8.8261	4.9012	12.3738	49.8358	6.2234
P014	28-31	36.71	17.6221	7.9117	3.7654	9.7642	42.1415	3.5691
P015	>=32	35.43	34.9916	23.0624	16.053	57.5203	332.091	154.3071
P015	>=32	35.57	35.8551	23.4658	15.6868	55.2143	339.2748	166.6582

P015	>=32	35.71	29.23	18.9232	12.1929	37.3788	230.3458	96.8233
P015	>=32	35.86	28.0201	17.9374	11.1453	35.2804	213.7326	86.6208
P016	>=32	33.29	19.1648	9.6694	5.6328	18.7877	59.3359	6.4364
P016	>=32	33.43	19.9268	11.1775	6.8098	17.9303	81.0556	9.8279
P016	>=32	33.57	43.5594	23.8403	14.9364	76.535	385.7921	42.4141
P017	>=32	33.86	26.7158	15.2037	7.7839	41.5127	162.3778	14.324
P017	>=32	34	28.6736	16.7072	7.6307	46.3808	213.6418	12.7858
P017	>=32	34.29	30.9975	18.3076	9.4968	55.2649	247.0844	22.1926
P017	>=32	34.43	24.8374	15.8812	8.009	40.5463	181.7246	15.7493
P017	>=32	34.57	26.8184	16.7312	10.8177	41.8612	182.3324	31.0346
P017	>=32	34.71	25.3473	15.3998	7.7034	42.6399	165.2036	15.3555
P017	>=32	35.14	18.2417	11.0463	5.7385	21.7943	83.5822	8.339
P017	>=32	35.29	19.9455	11.6939	5.8723	23.8642	96.4704	8.3186
P017	>=32	36.71	19.4376	10.5327	5.27	19.6098	76.9821	7.0366
P017	>=32	36.86	21.8972	11.1445	5.508	20.9409	89.2141	7.5079
P018	>=32	32.86	21.659	10.6284	4.3624	20.5913	75.7382	16.2852
P018	>=32	33	19.6927	9.1171	3.542	15.3723	56.3583	11.7543
P018	>=32	33.14	24.69	10.8349	4.387	22.7209	78.7353	17.8908
P018	>=32	33.29	20.4479	9.4961	3.8689	16.2948	59.8439	15.8648
P018	>=32	33.71	23.2334	11.7996	5.1444	23.9154	91.2161	27.5141
P018	>=32	33.86	21.2555	11.1507	4.928	21.4027	83.9326	22.6706
P018	>=32	34	24.0399	11.802	5.7363	23.2194	89.9797	25.5207
P018	>=32	34.14	21.7229	11.2276	4.8293	24.0842	84.3474	19.4485
P018	>=32	34.29	22.1954	11.211	4.9848	21.2733	83.4326	21.2418
P018	>=32	34.43	21.4586	11.3931	5.0505	20.5439	89.1209	22.4694
P018	>=32	34.57	18.3247	9.3976	4.0615	13.4502	59.3752	14.3953
P018	>=32	35.29	19.1999	9.6525	4.3326	14.0341	64.708	15.9133
P018	>=32	35.43	18.819	8.7939	3.3736	13.7552	55.7472	10.9644
P018	>=32	35.57	17.7689	8.7339	3.3982	12.3764	53.7797	10.8445
P018	>=32	35.71	19.9996	9.7632	4.0273	14.2701	67.9698	14.4159
P019	28-31	30.29	12.645	5.9776	3.7917	5.1605	24.082	6.684
P019	28-31	30.43	13.5099	6.5712	4.3087	5.3263	27.9731	8.2285
P019	28-31	30.71	14.8135	8.0784	5.3391	7.8595	40.1183	15.04
P019	28-31	30.86	14.7287	8.4226	4.889	12.7775	50.8339	14.4865
P019	28-31	31	14.192	7.5258	4.596	8.0352	36.9991	10.6497
P019	28-31	31.14	15.1392	8.4564	5.1294	8.816	46.8636	14.9105
P019	28-31	31.29	13.8291	8.5821	5.1063	10.8246	52.0856	15.5628

P019	28-31	31.86	12.5143	6.4101	3.773	6.4076	25.6261	7.8585
P019	28-31	32	14.06	6.4613	4.055	6.3145	25.2522	8.1873
P019	28-31	32.14	12.8977	6.6359	4.2725	6.3242	26.7843	8.4219
P019	28-31	32.29	12.2863	6.4921	4.4444	6.1412	25.3922	8.722
P019	28-31	32.43	12.0386	6.1795	3.926	5.8841	22.6491	6.9158
P019	28-31	33.57	13.9406	9.1003	5.8534	10.2175	54.5195	19.4153
P019	28-31	36	22.1778	11.1014	5.6557	15.7235	87.9296	20.3297
P019	28-31	36.14	18.8332	9.2547	4.615	10.2665	61.5937	12.1208
P019	28-31	36.29	22.5833	10.1371	5.1317	13.2325	73.7344	14.6924
P019	28-31	36.57	25.9126	12.3649	6.409	17.6324	93.716	25.8363
P019	28-31	36.71	21.3961	10.2856	5.5555	13.7884	71.6135	17.5211
P019	28-31	36.86	21.6955	10.6659	5.6829	14.2671	78.1956	18.7513
P020	>=32	36	33.7384	18.1067	12.925	44.7627	195.4938	38.9507
P020	>=32	36.14	37.8038	19.9622	12.2226	74.8532	252.6452	30.2423
P020	>=32	36.29	40.165	22.4845	14.9102	79.6292	323.3186	50.6443
P020	>=32	36.57	37.0789	23.1163	13.4569	70.3242	369.5979	45.5425
P020	>=32	36.71	38.188	23.2908	13.4341	73.04	383.8633	47.8746
P020	>=32	36.86	36.7798	22.7504	13.0264	67.8429	369.1374	45.3408
P021	>=32	32.86	17.5438	6.8287	3.7705	9.8202	28.7428	3.0179
P021	>=32	33	20.1876	8.0833	4.7658	11.9985	40.3634	4.9661
P021	>=32	33.14	21.3757	8.8807	4.7294	15.7291	50.2382	4.6791
P021	>=32	33.29	22.5877	10.9319	6.5087	18.3654	74.8069	9.6422
P021	>=32	33.71	34.222	16.744	10.9431	38.4754	171.0184	26.8784
P021	>=32	33.86	36.7616	18.3171	12.3729	42.1108	212.827	33.1507
P021	>=32	34	33.6377	16.822	10.9233	39.0312	180.6154	27.7733
P021	>=32	35	30.8538	16.4145	14.2655	27.1995	148.8558	44.4596
P021	>=32	35.14	32.7706	15.4583	12.5091	27.4517	138.8045	33.9528
P021	>=32	35.29	28.8069	14.0199	12.4362	20.7588	106.4107	34.04
P021	>=32	35.57	31.8949	17.0866	16.7485	24.9661	146.29	64.5345
P021	>=32	35.71	29.5465	15.4101	15.5441	20.8207	118.5714	51.7621
P021	>=32	35.86	28.3425	14.9502	13.7826	21.2216	114.0768	48.4477
P021	>=32	36.14	27.1237	13.7455	12.6532	18.3689	102.5843	38.5641
P022	>=32	32.29	12.6112	6.5055	3.7838	9.5017	27.3082	3.1038
P022	>=32	32.43	17.7519	8.9013	4.1172	14.2182	55.8494	3.3898
P022	>=32	32.57	30.333	16.072	8.2089	37.7017	181.5938	14.9098
P022	>=32	33	36.0654	19.0995	9.2978	60.2659	255.4065	18.0697
P022	>=32	33.14	38.5684	19.7746	9.2982	61.0132	275.4075	17.2826

P022	>=32	33.29	37.7066	21.8131	10.8169	67.2648	341.6737	24.7536
P022	>=32	33.71	48.5526	28.2249	15.9401	107.9032	536.8437	61.5586
P022	>=32	33.86	48.4746	27.51	15.6607	94.6144	507.7729	54.7641
P022	>=32	34	44.2009	27.2945	16.1738	92.342	488.9923	68.9838
P022	>=32	34.14	44.4077	27.9686	16.2514	98.3857	510.8107	67.5553
P023	28-31	31.57	18.9959	9.5579	6.4458	13.3081	55.0812	10.7618
P023	28-31	31.71	21.9971	12.2332	7.6405	19.3285	94.9668	15.1993
P023	28-31	31.86	19.1975	11.347	6.8756	16.3549	80.8981	11.7721
P023	28-31	32	19.3753	10.9416	6.5559	16.2758	76.127	10.3913
P023	28-31	32.29	21.1271	12.4783	8.1109	18.9109	95.8045	16.3438
P023	28-31	32.43	21.345	12.5151	8.2457	19.665	93.6804	18.1989
P023	28-31	32.57	22.5954	14.2287	9.3104	24.5054	122.822	22.417
P023	28-31	32.71	21.3104	13.2985	8.6559	21.4023	107.53	19.0068
P023	28-31	33	22.945	14.5619	9.1252	22.6211	129.9818	21.2993
P023	28-31	33.14	19.884	12.9154	8.0517	17.6132	103.5207	18.222
P023	28-31	33.29	17.9603	11.3577	6.8355	14.4695	81.7797	12.4675
P023	28-31	33.43	17.0559	10.6703	6.065	14.2525	74.8558	9.2781
P023	28-31	33.71	17.8769	11.7174	6.8378	14.2446	89.9964	12.428
P023	28-31	33.86	19.9651	12.5387	7.4966	18.3291	101.7418	14.9325
P023	28-31	34	19.1955	11.9978	7.2975	17.6304	91.6989	14.3096
P023	28-31	34.29	20.4291	12.3107	6.9816	17.7959	100.0755	13.0556
P023	28-31	34.43	21.1103	13.3048	7.7861	23.2534	115.1686	16.0931
P023	28-31	34.57	23.2662	14.3859	8.5101	24.5521	136.5167	18.7498
P023	28-31	34.71	21.2577	12.9469	7.4318	20.1206	110.6399	15.3635
P023	28-31	35	20.3496	12.0613	6.686	17.6576	97.912	12.3449
P023	28-31	35.14	19.0359	12.0869	7.177	17.5325	95.8886	15.1161
P023	28-31	35.29	19.4885	12.449	7.5144	18.171	101.4545	15.9186
P023	28-31	35.43	21.6295	14.1411	9.2371	19.9427	124.8197	23.4719
P023	28-31	35.86	23.6485	14.7637	8.9935	22.7255	143.4667	23.9351
P023	28-31	36	25.789	15.0151	8.2504	26.19	157.2542	18.3653
P023	28-31	36.14	28.9009	17.5906	10.3434	32.7465	214.2959	28.1246
P023	28-31	36.43	25.7331	16.1133	8.8687	28.3086	184.3684	20.7464
P023	28-31	36.57	27.7308	16.6481	9.9759	26.4992	183.7348	28.4306
P023	28-31	36.71	25.5669	16.7615	10.4537	29.2597	184.2061	30.7621
P023	28-31	36.86	27.7748	17.6493	10.7053	31.8091	205.7435	32.1616
P024	28-31	33	18.787	8.1613	3.4144	15.8629	45.1745	3.0163
P024	28-31	33.29	16.6727	8.0577	4.0708	12.7918	43.1434	4.7449

P024	28-31	33.43	16.2909	8.221	3.4072	14.9485	47.0501	3.2803
P024	28-31	33.57	16.193	8.4005	3.2218	15.8875	51.1267	2.7698
P024	28-31	33.71	20.3118	9.9586	3.9939	21.4413	70.3691	4.1848
P024	28-31	33.86	19.1282	8.7082	3.1716	16.8013	53.1619	2.6788
P024	28-31	34.14	20.472	9.7925	3.9251	18.422	69.3808	3.925
P024	28-31	34.29	20.8253	10.8578	4.2559	23.365	95.1795	4.3662
P024	28-31	34.43	18.7228	9.5252	3.9146	16.0609	71.0631	3.8146
P024	28-31	34.57	20.7798	10.5489	4.126	19.7216	85.7925	4.1703
P024	28-31	34.71	20.6851	10.1551	4.4269	15.8877	77.1397	5.3178
P024	28-31	35.14	20.5217	10.798	4.6797	16.1732	88.6579	6.5041
P024	28-31	35.29	21.334	11.1223	4.5489	17.6264	95.8406	5.4677
P024	28-31	35.43	20.0043	10.9564	4.5503	15.225	94.7077	5.787
P024	28-31	35.57	19.7122	10.423	4.322	13.381	83.7151	5.232
P024	28-31	35.86	19.7195	10.5547	4.1076	14.6094	89.8102	4.4165
P024	28-31	36	17.8178	9.5638	4.093	12.9919	69.6178	4.7308
P024	28-31	36.14	19.9087	9.798	3.9551	12.1281	74.5655	4.3525
P025	<28	31.86	20.6039	10.2546	6.3304	15.8614	64.6837	11.1155
P025	<28	32	24.5768	13.2523	9.1534	19.2828	102.5746	26.2518
P025	<28	32.14	19.7048	9.468	5.4608	14.6941	56.4523	8.7174
P025	<28	32.29	19.3714	10.7868	6.7625	22.2001	72.4826	14.4329
P025	<28	32.86	17.1985	7.6197	3.8224	10.5056	39.2537	3.899
P025	<28	33	16.8282	8.5221	4.4777	12.5661	49.5873	5.3392
P025	<28	33.14	17.4764	8.7916	4.9289	11.7914	50.8146	6.9924
P025	<28	33.29	18.7153	10.2544	5.357	17.7525	71.2273	7.5914
P025	<28	33.71	18.5382	9.5971	6.4796	12.3619	54.6352	12.0847
P025	<28	33.86	17.6376	8.8147	5.2295	13.2439	52.4371	7.3319
P025	<28	34	18.9017	8.8873	4.6651	13.6431	53.11	5.4927
P025	<28	34.14	18.4952	10.4243	6.2922	16.0406	74.7071	10.8837
P026	>=32	33.43	28.8154	14.8755	10.4941	33.2853	133.1688	30.8581
P026	>=32	33.57	29.4536	15.8181	11.4959	29.026	144.3813	39.5381
P026	>=32	33.71	26.0868	15.2799	11.2863	27.0293	126.4344	37.3722
P026	>=32	33.86	25.2922	15.1241	12.2952	24.5858	114.2387	46.3375
P026	>=32	34	22.0998	13.2231	9.709	19.6266	90.0269	28.9607
P026	>=32	34.43	31.6918	16.9654	11.4663	30.599	161.3696	39.724
P026	>=32	34.71	30.1922	16.5399	12.0954	26.1405	142.6594	44.807
P026	>=32	34.86	27.9614	16.1681	11.3888	25.8882	142.692	38.4455
P026	>=32	35.29	24.9839	15.4064	10.105	24.7322	146.3857	31.1205

P026	>=32	35.43	21.4816	13.6193	9.1323	21.1337	110.773	23.9132
P026	>=32	35.57	27.42	16.1845	10.6415	30.1242	157.8	34.3395
P026	>=32	35.71	23.9426	14.3124	8.7657	25.1343	130.3718	22.2499
P026	>=32	36.29	26.9932	15.0689	8.8622	26.3757	150.7888	24.0434
P026	>=32	36.43	23.0681	12.8783	7.3763	19.5136	109.311	16.7515
P026	>=32	36.86	28.1312	14.7817	8.9987	28.1762	139.0231	24.9011
P027	>=32	32.43	15.5859	7.6784	3.4151	12.406	40.0877	2.7351
P027	>=32	32.57	16.3965	7.777	3.0648	12.6943	42.4139	2.2336
P027	>=32	32.71	16.1172	8.2366	3.3868	13.8771	47.1725	2.6869
P027	>=32	32.86	15.7982	8.4306	3.505	14.3808	49.8234	2.8898
P027	>=32	33	17.1496	9.7851	4.4747	16.9895	65.7476	5.593
P027	>=32	33.14	18.2867	10.4586	4.9412	16.8348	75.0452	6.9674
P027	>=32	34.14	19.3889	10.9852	5.0809	19.1514	82.2131	7.5626
P027	>=32	34.29	19.6244	10.6067	4.8505	18.5206	77.1461	7.3272
P027	>=32	34.43	19.4174	10.6824	5.0242	19.0309	78.3598	7.728
P027	>=32	34.57	17.7289	9.9725	4.602	14.7703	68.1518	6.557
P027	>=32	34.71	16.7078	9.2185	4.018	12.9883	59.5544	4.9337
P027	>=32	36.43	16.7809	7.9603	3.5235	9.4761	44.2233	3.3809
P027	>=32	36.57	17.236	8.4097	4.0615	10.0685	47.8912	4.7867
P027	>=32	36.71	15.6854	8.0578	4.3215	8.5175	42.5378	5.3449
P027	>=32	36.86	19.2449	10.0469	5.2112	12.5125	68.7363	7.5583
P028	28-31	28.14	11.4976	3.9486	2.9453	2.716	9.2029	2.2948
P028	28-31	28.43	16.1063	6.7128	4.5498	7.4032	28.156	5.1969
P028	28-31	28.57	14.797	6.4413	3.3418	7.5155	28.1112	2.73
P028	28-31	28.71	13.9837	6.7593	3.7269	7.9354	31.1576	3.4614
P028	28-31	28.86	15.0159	7.3779	3.7153	10.7343	40.1963	3.2762
P028	28-31	29.14	17.4585	9.4534	5.9343	12.7572	59.9732	9.1092
P028	28-31	29.29	17.5871	9.1591	4.5119	13.4657	59.3864	4.3379
P028	28-31	29.43	17.5178	9.2899	4.4382	13.2045	62.0916	4.2218
P028	28-31	29.71	15.6453	8.2957	4.0061	10.2997	51.4007	3.3741
P028	28-31	29.86	14.3524	7.9533	3.7798	9.7429	46.2045	3.1917
P028	28-31	30.14	13.5861	8.0444	3.5451	12.1139	51.013	2.8583
P028	28-31	30.29	14.8941	8.8327	3.8222	15.5383	65.3449	3.3245
P028	28-31	30.43	13.9255	7.5732	3.4809	8.9773	42.2881	2.8566
P028	28-31	30.57	15.0778	8.1449	4.0399	9.7133	46.9361	3.8781
P028	28-31	30.71	15.1052	8.1875	4.0473	9.2352	47.5033	3.6178
P028	28-31	30.86	17.4906	10.3835	4.2679	14.248	81.88	4.083

P028	28-31	31	17.1178	10.4362	4.145	13.5905	82.775	3.7632
P028	28-31	31.14	15.3427	9.1715	3.6587	10.8407	63.8358	3.0263
P028	28-31	31.29	17.5423	10.6982	4.43	13.5186	87.279	4.4508
P028	28-31	31.43	19.9634	12.0482	4.9104	16.2523	110.4992	5.2785
P028	28-31	31.71	12.8016	6.808	3.0778	6.4016	33.8056	2.1054
P028	28-31	31.86	14.0778	7.595	3.4571	7.6457	42.9563	2.7155
P028	28-31	32.14	18.8026	10.8942	5.1188	15.0968	85.8723	6.1346
P028	28-31	32.29	18.2846	10.0813	4.4331	13.818	73.7275	4.6676
P028	28-31	32.43	15.9621	8.7766	4.269	8.5853	54.7244	4.532
P028	28-31	34.86	17.7462	9.9496	3.9671	12.5132	75.1909	3.667
P028	28-31	35	18.0164	10.6304	4.0334	17.0287	88.0896	3.5409
P028	28-31	35.14	15.8226	9.186	3.5803	10.4282	66.3772	2.9899
P028	28-31	35.43	19.1511	10.5171	4.0712	13.8287	84.9883	3.8467
P028	28-31	35.57	16.4491	9.2432	3.6963	10.3433	64.2869	3.391
P028	28-31	35.71	17.0545	9.9293	4.0668	13.0494	73.6519	4.1721
P028	28-31	36	15.1792	9.7268	4.725	9.8661	69.2178	6.1553
P028	28-31	36.14	17.2136	9.8855	3.6962	12.1973	73.6689	2.9689
P028	28-31	36.29	18.7744	10.3636	3.9212	12.6753	80.9827	3.4344
P028	28-31	36.43	16.8161	9.576	3.9059	10.9554	67.69	3.7333
P028	28-31	36.71	28.4877	15.8136	7.2941	31.241	182.8549	12.9719
P028	28-31	36.86	23.6316	14.614	6.3908	26.6629	154.601	9.4938
P029	>=32	34.14	32.9701	17.2864	9.5509	47.7199	208.5317	15.6019
P029	>=32	34.29	38.809	20.1905	10.4187	66.611	285.8235	20.8283
P029	>=32	34.43	33.1503	18.9786	9.4518	58.7217	255.8625	21.0873
P029	>=32	34.57	34.8811	20.1762	10.0069	59.552	292.2119	23.1338
P029	>=32	35	31.3427	17.8339	10.0549	42.1468	226.8603	25.5347
P029	>=32	35.14	27.5912	16.5482	8.6208	35.2055	192.7037	16.1376
P029	>=32	35.57	31.1111	18.2836	10.4131	42.865	236.0376	26.7299
P029	>=32	35.71	32.8259	16.4564	8.6578	40.5364	180.472	18.3099
P030	>=32	33.86	17.1691	6.9629	3.2457	10.02	34.6582	2.5759
P030	>=32	34	16.0311	7.8982	3.992	10.7892	43.0621	3.9237
P030	>=32	34.14	16.7362	8.2542	4.4	11.4661	45.8669	4.7469
P030	>=32	34.43	25.0505	11.6116	7.0893	22.5254	90.9731	10.4501
P030	>=32	34.57	19.0954	9.727	5.0948	17.1252	62.0312	6.444
P030	>=32	34.71	17.3893	9.4218	4.9314	14.7343	60.6847	5.7531
P030	>=32	34.86	15.9285	8.259	4.3008	10.7035	47.5248	4.8413
P030	>=32	35	17.4549	8.6664	4.8429	11.9302	49.0685	6.1725

P031	>=32	35.71	19.9855	10.9525	6.5101	17.7296	78.5288	10.9788
P031	>=32	35.86	18.7632	9.8812	5.6593	16.4081	64.2848	8.6329
P031	>=32	36	18.1382	9.4005	5.0929	13.5303	60.4738	7.2668
P031	>=32	36.14	19.4839	10.635	6.3078	18.0877	72.7623	10.8848
P031	>=32	36.29	16.6699	9.2232	5.2355	12.4252	56.1086	7.2059
P032	>=32	32.29	18.2333	8.6076	5.3883	11.8661	47.9533	7.4546
P032	>=32	32.43	21.9927	10.9428	5.3239	18.5465	83.3295	6.23
P032	>=32	32.57	18.5877	10.2415	5.1114	15.0748	74.5277	5.5232
P032	>=32	32.86	19.7467	9.7087	5.1495	13.2952	62.8999	6.2552
P032	>=32	33	19.3212	10.7003	5.5432	14.7799	78.1154	7.3934
P032	>=32	33.14	18.0419	9.9816	5.0009	13.669	69.401	5.7924
P032	>=32	33.29	24.7891	15.6998	8.8766	27.5587	170.6719	17.5828
P032	>=32	33.57	31.6175	19.2838	11.7583	43.3289	249.9625	34.2337
P032	>=32	33.71	30.1477	19.1687	11.4396	40.8788	240.9493	33.1961
P032	>=32	33.86	27.6966	16.9634	9.9864	30.4926	188.9804	24.549
P032	>=32	34	27.4901	18.6639	11.9699	36.6448	218.1388	36.3592
P032	>=32	34.29	27.0183	16.8202	9.5382	32.6589	190.8305	23.2088
P032	>=32	34.43	23.8423	15.1439	7.9932	26.0493	155.077	15.918
P032	>=32	34.57	24.4508	15.4186	8.1072	28.263	161.4011	16.4352
P032	>=32	34.71	25.1707	15.2918	7.722	25.6438	162.6365	14.2198
P032	>=32	35	25.7135	17.1078	9.3373	32.4434	199.4481	21.0596
P032	>=32	35.14	27.5993	17.907	9.8136	34.3628	226.6287	24.2759
P032	>=32	35.29	25.8536	15.513	7.6457	29.3752	167.948	13.2697
P032	>=32	35.57	24.0311	15.0067	7.3566	28.3033	161.3695	12.8438
P032	>=32	35.71	23.9522	14.0554	6.4463	23.0698	140.5392	8.8891
P032	>=32	35.86	22.8465	13.1368	6.1386	21.6118	124.728	8.0531
P032	>=32	36	25.5275	16.4715	7.9661	33.2996	204.6131	14.2943
P032	>=32	36.29	28.7781	19.7234	9.4574	47.9943	287.7139	20.8889
P032	>=32	36.43	26.5398	17.2723	8.7195	35.4467	215.4104	18.1473
P032	>=32	36.57	27.0997	17.524	9.2258	35.6871	215.3511	20.466
P032	>=32	36.71	30.1809	17.8123	8.2752	40.2443	229.3249	13.9908
P033	>=32	34.86	23.5362	11.8731	8.4028	20.8212	82.6958	16.7474
P033	>=32	35	27.9965	13.7771	8.913	34.7622	115.5053	16.9403
P033	>=32	35.14	38.3107	19.9094	14.5922	64.9164	235.856	46.3509
P033	>=32	36.14	28.747	16.2781	11.9129	29.3589	163.423	40.9364
P033	>=32	36.29	27.8645	16.3784	11.5747	28.5348	160.9387	37.8756
P033	>=32	36.43	23.6523	14.0611	9.9333	21.3147	120.0112	27.9251

P033	>=32	36.86	26.4084	14.3985	9.9561	24.0486	125.7594	28.0827
P034	>=32	34.86	18.1723	12.1609	9.1651	16.9261	87.159	23.505
P034	>=32	35	20.9904	13.0562	7.5514	22.588	108.3262	12.2544
P034	>=32	35.14	18.7732	13.3385	7.6013	19.1979	110.82	11.8479
P034	>=32	35.29	19.164	14.5255	8.5449	21.7999	127.6305	19.7842
P034	>=32	35.43	23.1281	16.6017	9.7464	29.5256	170.2096	21.2836
P034	>=32	35.71	21.4105	15.2177	8.6279	29.6707	146.2448	20.8394
P034	>=32	35.86	20.6584	14.9964	8.2446	31.9063	148.4417	19.7838
P034	>=32	36	19.3019	14.0841	7.5542	25.1733	130.01	17.1035
P034	>=32	36.14	19.6123	13.5043	7.2607	25.5055	121.0466	15.6662
P034	>=32	36.29	22.9636	15.544	7.9983	30.727	167.0038	19.2312
P035	28-31	28.57	6.4217	2.619	2.7734	1.0484	2.427	2.4656
P035	28-31	28.71	6.125	2.5614	2.9343	0.8606	2.2294	2.7966
P035	28-31	28.86	9.1094	3.2034	2.0552	2.3483	6.052	1.3947
P035	28-31	29.29	12.4964	4.1143	2.4514	3.8605	9.8232	1.8311
P035	28-31	29.43	12.1665	4.2335	3.1632	3.7826	9.4863	3.0289
P035	28-31	29.57	13.5338	4.7903	2.6891	4.5827	13.8309	2.0152
P035	28-31	29.86	14.0374	5.7305	2.997	6.3044	20.8628	2.5165
P035	28-31	30	14.5317	5.8486	3.0433	7.1713	21.3263	2.598
P035	28-31	30.14	15.2898	5.6896	2.7604	7.437	19.9933	2.1177
P035	28-31	30.29	15.4519	5.531	2.8192	6.4531	18.8824	2.2624
P035	28-31	30.57	15.0308	6.6508	3.9013	7.2086	26.7969	4.6493
P035	28-31	30.71	15.7689	6.2776	3.0477	8.2987	24.6477	2.6964
P035	28-31	30.86	13.9205	5.5679	2.5057	6.2845	20.2624	1.778
P035	28-31	31	13.6475	5.8049	2.7891	6.3378	21.7831	2.3646
P035	28-31	31.29	14.8029	6.343	3.179	6.5569	25.6899	3.1686
P035	28-31	31.43	13.4937	5.5738	2.4371	6.2118	20.4017	1.8248
P035	28-31	31.57	11.3538	4.6781	2.0946	4.6401	14.1905	1.5033
P035	28-31	31.71	10.5785	4.7733	2.5055	3.813	14.3186	2.3063
P035	28-31	32	11.2915	5.9496	2.4711	6.3228	25.4752	1.9866
P035	28-31	32.14	12.2281	5.2347	2.7268	4.9485	17.7113	2.5132
P035	28-31	32.29	11.9574	5.3481	2.7651	5.2386	19.3921	2.3554
P035	28-31	32.43	12.956	5.8629	3.4887	6.2429	23.1754	3.9641
P035	28-31	32.71	14.7177	7.4679	3.5646	9.9004	39.5321	3.8421
P035	28-31	32.86	14.642	7.5685	3.7733	11.0984	39.5613	4.2143
P035	28-31	33	16.3098	7.6766	3.5373	10.4471	41.4311	3.6956
P035	28-31	33.14	15.0295	6.8296	2.8963	8.9073	31.6957	2.4405

P035	28-31	33.57	15.0546	6.8601	2.7212	9.3143	31.8468	2.16
P035	28-31	33.71	16.0331	7.3892	3.3109	10.1765	39.0898	3.1856
P035	28-31	33.86	14.5609	6.9561	3.1872	8.6132	33.2268	3.0166
P035	28-31	34	14.2498	7.5189	4.7499	9.521	36.3344	7.3656
P035	28-31	34.43	15.466	7.9368	3.1502	13.8389	45.3429	2.7966
P035	28-31	34.57	14.7133	7.5842	3.9574	10.8872	37.4772	4.9256
P035	28-31	34.71	16.9744	8.3051	4.3146	12.0926	47.0086	5.3853
P035	28-31	34.86	16.7718	7.8719	3.6441	11.2833	42.458	4.0059
P035	28-31	35.71	21.7286	10.3038	4.2297	14.7418	78.1232	4.9967
P035	28-31	35.86	21.001	11.9457	7.8787	16.5508	89.2105	18.694
P035	28-31	36.57	24.0397	13.8436	6.8201	26.6695	135.815	12.2388
P035	28-31	36.71	23.4707	13.4056	5.9747	25.5683	130.8642	9.0763
P036	28-31	31	18.0672	9.1836	5.3052	16.6851	54.1702	7.6657
P036	28-31	31.14	23.4615	11.4352	5.6944	27.4356	92.3258	7.7857
P036	28-31	31.29	19.9722	10.2439	4.6883	21.1434	76.6722	5.3674
P036	28-31	31.43	19.8105	10.269	4.7136	21.9437	75.9489	5.3409
P036	28-31	32	18.2556	8.5281	3.6309	17.8282	50.4674	3.3575
P036	28-31	32.14	16.3073	8.4448	3.3161	16.0595	52.9378	2.4329
P036	28-31	32.29	17.2784	7.1194	2.9198	14.0471	37.0969	2.2167
P036	28-31	32.43	15.6866	6.9048	2.8093	12.8752	32.7215	2.0591
P036	28-31	32.86	14.3468	6.805	2.9082	12.5705	37.2538	2.379
P036	28-31	33	14.2009	7.0291	2.8609	15.2005	36.9115	2.1856
P036	28-31	33.14	14.6476	7.7362	3.4882	13.7744	45.3696	3.1397
P036	28-31	33.29	15.2187	7.1197	3.214	11.1051	35.2671	2.7037
P036	28-31	33.71	15.3111	7.5071	2.9001	12.9909	44.0838	2.0284
P036	28-31	33.86	14.3135	7.3949	2.7905	10.5833	42.1786	1.8391
P036	28-31	34	14.3059	8.0352	3.0186	14.032	53.1252	2.0345
P036	28-31	34.29	14.2125	6.7712	2.8388	9.4617	33.6786	2.1882
P036	28-31	34.43	15.3458	6.5842	2.4627	9.3331	33.8165	1.4903
P036	28-31	34.71	14.2736	6.4895	2.4204	8.1427	31.4322	1.506
P036	28-31	34.86	15.1752	7.4222	2.9983	12.0737	43.7342	2.045
P036	28-31	35	15.2185	7.0513	3.4807	8.7329	34.6466	3.2796
P036	28-31	35.14	16.3825	8.1987	3.8195	10.8028	49.8446	3.6282
P036	28-31	35.29	15.2733	7.9717	3.5359	10.5688	46.9883	3.2804
P036	28-31	35.57	15.7537	9.838	3.9733	20.2406	85.135	8.3112
P036	28-31	35.71	14.3918	7.4675	2.9376	10.7786	47.8251	4.2843
P036	28-31	35.86	15.3603	7.5857	2.8048	8.7641	47.227	3.561

P036	28-31	36	15.8637	7.0472	2.7979	8.0862	38.4731	3.4891
P036	28-31	36.14	15.2871	8.5056	3.606	10.3065	62.0648	5.6366
P036	28-31	36.43	17.599	8.6945	3.8146	11.8882	60.3684	6.243
P036	28-31	36.57	21.6814	15.366	14.3162	21.1781	133.8694	84.6864
P037	>=32	33.57	23.0677	9.5611	6.0722	14.9199	59.6742	14.1037
P037	>=32	33.71	24.9226	11.5632	7.8909	21.4563	83.5406	27.0718
P037	>=32	33.86	20.5682	10.0475	6.1303	16.1095	66.0563	16.9097
P037	>=32	34	20.8998	10.7321	5.7837	21.1998	79.0465	14.8573
P037	>=32	34.29	24.806	12.9049	6.8434	27.6698	118.3167	21.0908
P037	>=32	34.57	25.3407	14.0327	7.9466	32.6793	135.6947	28.8202
P037	>=32	34.71	19.9396	10.6642	5.542	21.1072	76.711	15.5639
P037	>=32	34.86	23.3386	11.5206	5.9478	22.4279	91.5091	18.9633
P037	>=32	35	20.8549	10.6172	5.356	19.0166	77.4276	15.3404
P037	>=32	35.14	22.7695	11.1082	5.2295	20.0885	87.9055	14.9174
P037	>=32	35.43	19.7307	9.3095	4.7089	14.0342	59.5297	12.0768
P037	>=32	35.57	22.2452	10.2774	5.3768	16.8043	72.8235	15.9772
P037	>=32	35.71	23.3248	10.5919	4.9299	19.0499	80.5795	13.4993
P037	>=32	35.86	24.8753	11.1617	5.5854	21.7181	86.8089	15.8171
P037	>=32	36	21.8377	9.6088	4.6757	16.3997	64.8997	10.7576
P037	>=32	36.29	24.3161	11.0109	5.9729	19.0637	84.9885	16.83
P037	>=32	36.43	23.251	10.2682	5.517	16.8801	73.4285	14.1734
P037	>=32	36.57	24.6502	10.5503	5.7402	15.6821	79.0513	16.2511
P037	>=32	36.71	20.4369	8.5911	4.6693	10.6574	51.466	10.6934
P038	>=32	33	11.3965	5.2992	4.4461	4.4101	14.137	5.2886
P038	>=32	33.43	14.6179	7.1746	3.6937	9.5691	34.9483	3.476
P038	>=32	33.57	14.346	7.7664	5.7049	8.5722	37.501	8.9082
P038	>=32	33.71	15.6122	8.5274	5.2064	11.8129	47.1729	7.1654
P038	>=32	33.86	18.7633	9.9009	5.3595	15.4186	69.1404	7.2887
P038	>=32	34.14	15.853	8.4851	4.3654	12.2262	49.9186	4.9858
P038	>=32	34.29	19.4765	9.9696	4.9884	19.888	68.3466	5.8627
P038	>=32	34.43	20.4022	10.7973	5.1422	22.2924	82.3317	5.9792
P038	>=32	34.57	21.7596	11.0489	5.3707	23.8713	83.8897	7.1671
P038	>=32	35	17.108	8.4657	4.255	12.955	49.2671	4.7112
P038	>=32	35.14	18.8169	9.1964	4.5817	14.9714	59.3236	5.2798
P038	>=32	35.29	16.5472	8.8961	4.4433	11.5725	56.7851	4.9107
P038	>=32	35.43	18.4882	9.4751	4.4924	15.1322	64.0799	4.7831
P038	>=32	35.71	18.1487	9.4035	4.5688	13.8853	62.5276	5.3655

P038	>=32	35.86	20.3501	10.485	4.9261	18.3004	79.0989	5.9233
P038	>=32	36	22.6997	11.2292	6.9496	17.5852	92.3665	12.5304
P038	>=32	36.14	21.2727	10.1805	5.8621	14.9832	69.7641	9.3814
P038	>=32	36.71	18.9768	9.6567	4.7685	14.2437	64.8916	5.8981
P038	>=32	36.86	21.6045	11.523	5.1086	18.3582	100.1778	6.1448
P039	>=32	35.71	33.9182	20.2547	13.4144	47.3159	233.8362	36.7139
P039	>=32	35.86	34.5582	21.1099	13.1663	57.6955	270.8571	39.9396
P039	>=32	36	32.658	19.0433	11.8047	47.5109	212.1603	35
P039	>=32	36.43	26.2592	16.331	8.5851	35.648	174.7843	19.299
P039	>=32	36.57	21.0479	13.2953	6.7276	23.5834	119.0175	12.0722
P039	>=32	36.71	24.0448	13.6673	6.9993	25.666	125.5018	12.5667
P039	>=32	36.86	25.0396	14.0178	7.4897	28.0542	131.3949	14.5989
P040	<28	26.57	11.6875	4.2021	3.6233	2.4281	8.684	4.0654
P040	<28	26.71	10.3928	3.5955	2.9135	1.8251	6.442	2.7045
P040	<28	26.86	9.4772	3.676	2.7242	2.1144	7.3311	2.2501
P040	<28	27	8.5817	3.8525	3.3405	2.1648	6.9611	3.3432
P040	<28	27.14	8.5008	3.6154	3.0945	1.965	6.1997	2.8252
P040	<28	27.57	9.79	4.8522	3.5911	3.5844	13.5549	3.635
P040	<28	27.71	12.1205	5.8227	3.832	5.5732	20.297	3.9159
P040	<28	27.86	16.3515	7.6853	5.4236	7.3816	34.675	7.6534
P040	<28	28.14	13.1952	6.3189	4.0724	5.4815	24.8047	4.3424
P040	<28	28.29	11.5912	5.0959	4.2827	3.2512	13.3689	5.1432
P040	<28	28.43	12.2464	5.3353	4.1116	4.4042	15.2141	4.7018
P040	<28	28.57	10.4345	5.1706	5.4109	3.0532	9.8493	8.5655
P040	<28	28.86	8.9991	3.7815	3.0851	2.348	6.6782	2.8234
P040	<28	29	10.0142	4.8058	3.2769	3.8046	13.3006	3.0059
P040	<28	29.14	9.1105	4.7128	2.981	3.8262	14.2125	2.6042
P040	<28	29.29	9.885	4.8914	3.6015	3.4126	12.6332	3.8277
P040	<28	29.43	9.6776	5.7062	5.1769	4.2162	16.4186	8.0874
P040	<28	29.86	11.5033	7.5971	7.8068	6.9954	24.1618	18.4393
P040	<28	30	11.3395	6.5741	4.3819	6.8947	28.3845	5.1727
P040	<28	30.14	11.8417	6.8894	5.8873	6.7414	24.8942	10.0341
P040	<28	30.29	12.3378	7.3532	4.5262	9.2055	38.7032	5.441
P040	<28	31.57	11.9929	7.07	4.4505	10.2277	32.091	5.7593
P040	<28	31.71	13.136	6.8613	4.7628	8.0772	27.4206	6.2912
P040	<28	31.86	13.0382	6.7963	4.0506	8.4338	29.2874	4.5169
P040	<28	32.29	12.8395	7.0657	4.1034	9.8306	30.6688	4.5762

https://doi.org/10.3390/xxxxx

P040	<28	32.43	15.6011	8.2345	5.2009	11.1995	39.3683	7.0748
P040	<28	32.57	15.6876	8.5817	5.5906	11.0315	44.2936	8.526
P040	<28	32.71	16.5185	8.1836	4.8743	11.5404	41.7994	6.4374
P040	<28	32.86	14.1674	7.8687	4.8731	10.2643	38.6406	6.3944
P040	<28	33.14	15.066	8.6144	5.0926	11.8998	46.5796	6.5332
P040	<28	33.29	17.2423	9.6479	6.2124	13.5311	55.4233	10.1302
P040	<28	33.43	17.0439	9.6431	6.1713	13.7255	55.0252	9.8245
P040	<28	33.57	16.9561	9.6883	6.2302	12.7764	56.2754	10.1018
P040	<28	33.86	15.9154	8.8793	5.2761	10.7166	50.5171	7.2223
P040	<28	34	15.605	8.2801	5.0822	8.6416	42.3276	6.7159
P040	<28	34.14	15.8533	9.0988	5.6192	11.409	51.2246	8.3517
P040	<28	34.29	15.5232	8.4579	5.0659	10.2526	45.964	6.724
P040	<28	34.43	16.47	9.4801	5.7046	13.3835	55.2416	8.4143
P040	<28	34.86	16.5868	9.8399	7.0236	10.9436	55.502	13.7602
P040	<28	35	16.126	9.079	5.2456	11.0637	54.1188	7.0014
P040	<28	35.14	15.9177	9.772	5.7752	12.867	62.0343	8.5409
P040	<28	35.29	18.4807	10.0462	5.8486	12.8806	65.0927	8.6832
P040	<28	35.57	18.7053	10.4734	6.2618	13.7872	69.1437	10.1541
P040	<28	35.71	18.0501	10.8715	6.4176	15.3587	75.1849	10.4685
P040	<28	35.86	17.0661	10.6148	6.5648	13.5339	70.1252	11.2094
P040	<28	36	17.2681	10.4569	6.3504	13.3081	67.8669	10.5654
P040	<28	36.43	17.8822	11.0078	6.6953	15.8285	76.4158	11.1844
P040	<28	36.57	18.1655	11.0383	6.5906	14.9969	78.7647	10.7682
P040	<28	36.71	18.0089	11.6898	6.8384	18.2734	87.676	11.3749
P040	<28	36.86	17.3251	11.0876	6.205	13.9266	81.408	9.3114
P041	>=32	35.71	23.5402	13.5242	8.8994	25.3979	112.525	18.4083
P041	>=32	35.86	27.8185	16.4195	12.5956	30.9429	154.8941	42.4674
P041	>=32	36	29.5954	18.0169	12.7777	38.74	194.2481	44.0039
P041	>=32	36.14	33.197	20.0802	15.7248	40.6137	230.0119	76.8837
P041	>=32	36.43	44.2733	26.7227	19.4392	80.9927	435.0333	107.4534
P041	>=32	36.57	31.4577	19.3987	14.4902	42.0808	218.9647	64.4134
P041	>=32	36.71	26.5492	18.138	13.1687	31.404	192.522	51.4658
P042	28-31	30.71	20.6718	8.6795	4.5007	23.1771	46.1892	8.3739
P042	28-31	30.86	18.8714	7.9089	3.8203	20.279	38.6252	6.3069
P042	28-31	31.14	18.7851	7.8189	3.6288	16.9158	40.4471	5.86
P042	28-31	31.29	15.5076	6.5568	3.1417	11.5977	27.7201	4.6058
P042	28-31	31.43	18.2029	8.013	3.427	16.7051	42.2082	5.9269

P042	28-31	31.57	17.4897	8.1066	3.5059	16.812	45.5996	6.8308
P042	28-31	31.86	20.0406	8.153	3.7352	17.3881	44.1503	7.1241
P042	28-31	32	21.0465	8.8959	4.3123	21.5372	51.4728	8.9692
P042	28-31	32.14	23.1415	8.8303	3.9793	21.7959	52.074	8.1085
P042	28-31	32.43	23.0758	9.0212	4.3693	22.1108	53.7776	9.2806
P042	28-31	32.57	23.8315	9.0535	4.9221	20.7835	53.7805	10.6895
P042	28-31	32.86	21.5436	9.5045	5.4065	22.2073	60.0818	11.9149
P042	28-31	33	21.4705	8.5115	4.4801	16.8625	49.0982	8.0869
P042	28-31	33.14	20.184	8.7536	5.0756	16.066	53.9076	9.8915
P042	28-31	33.29	21.5981	8.8149	4.6486	18.1226	53.2651	8.6662
P042	28-31	33.43	21.2173	8.6796	5.4989	14.7646	51.8443	11.4525
P042	28-31	34	24.4595	9.9037	6.9614	18.9733	62.8737	18.5644
P042	28-31	34.14	23.0965	9.734	7.0951	18.0381	61.3088	18.0935
P042	28-31	34.29	24.827	9.8937	7.6563	17.728	61.0496	20.5478
P042	28-31	34.43	26.8751	11.1847	8.5648	21.2842	78.4438	28.0651
P042	28-31	34.86	25.2261	10.6347	8.1488	19.7683	72.4523	23.8708
P042	28-31	35	25.1298	10.1037	5.8179	20.4179	71.814	14.6745
P042	28-31	35.14	24.2381	10.2585	5.5735	22.2316	74.1099	14.1487
P043	>=32	32.43	18.9766	10.6352	5.2582	16.8058	88.2158	13.4203
P043	>=32	32.57	19.7333	11.445	5.641	18.6486	100.783	16.2153
P043	>=32	32.71	19.1028	11.8269	7.1429	16.7041	101.5671	26.33
P043	>=32	33	19.7556	10.9811	5.1289	18.3511	91.368	13.6148
P043	>=32	33.14	19.6871	11.3306	5.2863	19.1367	98.5315	15.3878
P043	>=32	33.29	20.831	13.0666	6.8222	21.6976	132.6121	24.3413
P043	>=32	33.43	21.0856	12.2376	5.8188	21.5051	116.6084	17.876
P043	>=32	33.86	20.754	12.5785	6.0992	20.0695	125.4035	21.3268
P043	>=32	34	21.3773	12.5536	6.0717	20.0035	124.3949	20.5362
P043	>=32	34.14	20.9594	12.9105	5.7753	22.0544	134.7575	20.5037
P043	>=32	34.29	30.6293	19.8095	10.4457	46.6134	306.1354	61.1028
P043	>=32	34.43	24.2153	15.4785	7.2932	28.072	191.5887	30.8078
P043	>=32	34.86	26.389	16.0667	8.7647	30.9811	195.5335	40.5926
P043	>=32	35	21.2321	13.1656	6.0769	23.632	136.4329	20.5775
P043	>=32	35.14	21.0808	13.2906	6.275	22.6484	138.5062	21.8243
P043	>=32	35.29	19.9865	12.2245	5.714	19.7842	119.3521	18.2581
P043	>=32	35.57	25.4706	15.3309	7.0844	29.1906	186.8226	27.9518
P043	>=32	35.71	23.6349	15.5199	7.0058	27.6925	198.5436	30.0257
P043	>=32	35.86	23.6676	15.005	7.316	27.0037	175.9822	29.7066

P043	>=32	36	26.4151	15.9143	6.9977	34.6791	199.9574	28.123
P043	>=32	36.14	26.7276	15.5384	6.3835	34.5649	194.2398	23.2654
P043	>=32	36.43	24.7941	14.4027	5.7511	28.9836	165.674	19.3478
P043	>=32	36.57	23.6559	14.3794	5.9236	30.2899	168.3931	19.3881
P043	>=32	36.71	30.1088	18.2366	7.952	43.546	268.2807	33.4177
P043	>=32	36.86	30.0932	18.2569	7.632	43.5384	273.137	31.8737
P044	>=32	35.57	15.4234	9.1893	5.5254	13.003	57.3762	7.6107
P044	>=32	35.71	23.898	16.214	8.6469	33.7119	191.3561	15.338
P044	>=32	35.86	29.9448	18.1363	8.5712	38.0529	253.738	15.1018
P045	28-31	32.71	32.2678	17.3166	10.5726	41.891	181.0167	31.1668
P045	28-31	32.86	32.0667	18.0686	10.8105	46.1057	202.4027	30.4851
P045	28-31	33	31.3479	17.8658	10.9705	42.7146	202.3795	31.3637
P045	28-31	33.14	31.176	17.7494	10.9745	44.2164	194.7306	33.5717
P045	28-31	34.43	25.7812	13.1483	7.5794	25.354	109.6236	17.0499
P045	28-31	34.57	26.962	14.3831	9.0906	30.7558	120.4367	26.2557
P045	28-31	34.71	27.7907	14.6043	8.5475	27.7293	130.8899	22.3058
P045	28-31	35	32.2117	16.1103	9.9472	38.203	164.61	28.0138
P045	28-31	35.14	27.954	13.0234	6.8543	26.389	112.804	12.9077
P045	28-31	35.29	27.5634	12.7216	6.7302	24.8138	108.5848	13.6348
P045	28-31	35.43	28.0089	13.4079	7.1088	27.6698	121.0518	14.9717
P045	28-31	35.86	21.7823	11.2419	5.2604	19.3308	88.1135	8.1016
P045	28-31	36	23.6804	11.7831	5.6236	21.6709	97.3222	8.7716
P045	28-31	36.14	22.9295	11.729	5.633	21.6802	94.558	8.6054
P045	28-31	36.29	26.1742	12.1777	5.6611	23.0334	105.0221	8.3439
P045	28-31	36.57	20.9241	10.5552	4.963	19.8879	76.8593	6.155
P045	28-31	36.71	22.9087	10.844	5.3296	20.0196	79.4278	6.966
P046	<28	29.14	7.1945	3.5827	2.1381	2.8071	7.9045	1.254
P046	<28	29.29	7.6606	3.9864	2.4389	3.1219	9.9971	1.5266
P046	<28	29.43	8.0901	4.2536	2.1644	4.1831	11.4365	1.2242
P046	<28	29.71	8.9324	4.7066	2.8393	4.5885	13.6029	2.2074
P046	<28	29.86	9.522	5.2073	3.5599	5.1569	16.0766	3.8823
P046	<28	30.14	11.0409	6.0152	4.2122	6.9316	20.9575	4.8767
P046	<28	30.29	11.2984	6.6724	3.7309	7.5847	30.1283	4.1491
P046	<28	30.43	11.9532	6.3499	3.1547	7.6671	28.5494	2.9139
P046	<28	30.57	13.6705	7.0036	3.5871	10.5412	32.9234	3.7512
P046	<28	30.71	15.1671	6.6741	3.3287	10.8142	30.4914	2.9416
P046	<28	31.14	14.3972	6.7513	3.1045	8.4141	31.9555	2.7048

https://doi.org/10.3390/xxxxx

P046	<28	31.29	15.3625	6.6808	3.606	8.8814	28.5549	3.5528
P046	<28	31.43	12.5046	5.7606	2.5741	6.7721	23.6079	1.7373
P046	<28	31.57	15.5674	8.2369	3.8407	12.7833	47.3574	3.7509
P046	<28	31.86	14.3601	6.5508	3.1466	9.319	28.6709	2.6563
P046	<28	32	15.1814	7.3194	3.5361	12.4989	36.2697	3.2273
P046	<28	32.14	13.7421	7.3353	3.5743	10.9877	38.2361	3.2547
P046	<28	32.57	16.2695	7.7584	3.6635	11.9889	42.8958	3.3093
P046	<28	32.71	16.9935	7.9862	3.7127	12.0962	44.9397	3.328
P046	<28	32.86	17.0135	8.4896	4.0532	11.8935	53.7709	4.0478
P046	<28	33.14	17.0311	8.3426	4.1952	11.6434	50.2021	4.4032
P046	<28	33.29	17.8745	9.0574	5.0227	11.628	55.6561	6.7157
P046	<28	33.43	17.5166	8.9028	4.6701	12.7277	55.9006	5.7184
P046	<28	33.57	18.2604	10.1717	6.8194	14.5816	69.3856	13.1391
P046	<28	34	20.7867	10.9501	5.4866	19.055	85.5075	7.7244
P046	<28	34.14	22.0487	11.9724	6.066	18.434	106.064	8.8641
P046	<28	34.29	19.6656	10.6274	5.2865	17.2757	80.3361	7.1303
P046	<28	34.71	23.5054	13.3696	7.0624	23.2794	129.3094	11.4492
P046	<28	34.86	23.4655	13.364	6.9435	22.6011	132.9222	10.9919
P046	<28	35.29	26.5918	15.2687	9.1413	26.0944	156.6972	21.8882
P046	<28	35.43	28.7715	14.6449	7.8115	26.78	153.2329	14.3765
P046	<28	35.57	26.7101	14.267	7.5003	25.5575	144.2755	13.5999
P046	<28	36	30.5845	15.2299	7.5581	31.6004	168.2372	12.8393
P046	<28	36.14	28.6681	14.5717	7.3255	28.3683	155.6839	12.5769
P046	<28	36.29	28.5782	14.7154	7.6711	25.7222	153.0059	14.1438
P047	>=32	34.57	13.5812	6.1174	3.302	7.765	24.1438	2.6581
P047	>=32	34.71	16.6472	8.0845	4.1986	13.5605	43.3675	4.2513
P047	>=32	34.86	21.3008	11.0696	5.3307	21.7783	87.6689	5.9635
P047	>=32	35	24.6934	12.4452	5.7142	25.3672	113.1486	6.7426
P048	>=32	34.57	32.7739	17.6833	8.3015	60.8777	223.8695	11.0314
P048	>=32	34.71	30.2378	16.8856	7.272	51.6353	208.7354	8.8713
P048	>=32	35.29	19.8681	11.0305	4.6509	26.0876	84.4391	4.43
P048	>=32	35.43	25.7504	15.4026	7.0813	40.8254	171.4754	10.0602
P048	>=32	35.57	22.7055	13.5356	6.2053	29.6484	131.6902	8.5191
P048	>=32	35.71	27.2304	15.4845	7.3429	34.9776	175.6335	11.4989
P048	>=32	36.14	23.1663	14.5721	7.0156	27.5545	155.4804	11.1883
P048	>=32	36.29	24.2562	14.6266	6.6838	29.9816	159.3775	9.9281
P049	>=32	34.43	12.9993	4.8798	4.5973	3.6398	14.5737	3.2803

P049	>=32	34.57	18.4722	8.0481	5.7083	10.7621	43.6655	5.9663
P049	>=32	34.71	27.2991	14.23	10.1544	33.2571	124.035	22.5942
P049	>=32	34.86	27.3795	15.2943	10.2905	37.3576	147.4936	24.0828
P049	>=32	35.43	27.8795	15.9505	11.2333	27.6509	154.405	36.6944
P049	>=32	35.57	25.0584	12.982	7.6016	23.4514	112.1569	16.1675
P049	>=32	35.71	22.5957	12.6964	8.5709	19.3252	98.9347	21.6829
P049	>=32	36.14	18.9982	9.5226	4.6022	15.3988	62.4307	6.4318
P049	>=32	36.29	18.9312	9.3493	5.0726	12.9125	58.9054	7.8696
P049	>=32	36.43	26.6564	11.4485	5.5452	20.4232	94.4947	8.9325
P049	>=32	36.57	21.6711	8.3456	4.4636	10.6571	45.85	7.169
P049	>=32	36.86	22.0222	9.4825	5.0321	13.9914	55.2707	8.1402
P050	<28	30.57	15.82	9.7657	7.5116	11.6236	54.1226	15.5068
P050	<28	30.71	20.6035	13.1644	9.838	21.8564	99.7306	26.6199
P050	<28	30.86	18.6454	12.1339	9.4191	15.5957	82.0567	24.8319
P050	<28	31.29	19.6639	11.3854	7.5855	16.3969	81.1916	15.6075
P050	<28	31.43	18.2466	10.6749	7.5829	13.3347	67.4643	15.7107
P050	<28	31.57	16.472	9.1007	5.4952	10.6947	51.9571	8.3318
P050	<28	31.71	16.5832	9.4398	6.0446	12.4913	54.7211	10.3738
P050	<28	32.14	20.2723	12.2639	8.3366	19.5098	90.8464	18.8266
P050	<28	32.29	18.9404	11.4394	7.3798	16.1615	85.3839	14.6505
P050	<28	32.43	19.7951	11.7098	8.0597	14.0809	84.1573	18.2996
P050	<28	32.86	18.5968	11.375	8.8902	14.197	71.9865	21.7175
P050	<28	33	18.731	12.1817	10.0288	13.6115	78.5781	27.7358
P050	<28	33.14	22.371	14.6028	11.1064	16.9279	122.7501	35.0023
P050	<28	33.29	23.298	14.6716	12.1779	17.846	114.8366	42.2913
P050	<28	33.86	25.0861	15.3598	11.3354	24.3989	128.0511	36.0953
P050	<28	34	23.8416	13.6702	9.6602	20.3709	104.841	27.8928
P050	<28	34.14	21.6539	11.6339	7.6291	15.2981	79.0142	16.5049
P050	<28	34.29	22.2352	12.6895	8.5536	17.214	95.1341	20.5821
P050	<28	34.71	23.1476	14.1978	10.0703	23.1983	119.2729	25.4958
P050	<28	34.86	26.6818	16.1695	11.9941	26.997	150.0056	40.6087
P050	<28	35	22.1154	11.3486	6.7373	14.471	81.209	12.7112

The dataset contains 879 serial 24-hour HRV recordings obtained from 50 preterm infants. Each row represents one 24-hour HRV recording. Participants are identified using anonymized study identifiers to link repeated measurements within the same infant. The dataset includes gestational age group at birth, postmenstrual age at each recording, and six HRV indices (SDNNI, SDNN, RMSSD, VLF, LF, and HF). Direct

identifiers, recording dates, and other potentially identifying information are not included. Definitions of abbreviations are provided in the Abbreviations section of the main manuscript.

https://doi.org/10.3390/xxxxx
